# Jasmonic acid and nanoparticle elicitation of *L. cardinalis* – differentiating elicitor contributions in nanoparticle delivery strategies

**DOI:** 10.1039/d6ra03646e

**Published:** 2026-07-02

**Authors:** Rachel P. Sutherland, McKenna F. Clinch, Kristen Bruce, D. Trent Rogers, John M. Littleton, Bert C. Lynn, Stephen E. Rankin, Barbara L. Knutson

**Affiliations:** a University of Kentucky, Department of Chemistry 125 Chemistry/Physics Building Lexington KY USA; b University of Kentucky, Department of Chemical and Materials Engineering 177 F.P. Anderson Tower Lexington KY USA stephen.rankin@uky.edu bknut2@uky.edu; c Naprogenix™ UK-ASTeCC 145 Graham Avenue Lexington KY USA

## Abstract

Elicitation is an important strategy for producing plant secondary metabolites, which are responsible for a plant's highly dynamic chemical defense against environmental stressors such as UV light, predators, and pathogens. Elicitation strategies have recently employed nanoparticles as carriers to effectively deliver the elicitation agent through the cell membrane. This study examines the elicitation of secondary metabolites in *Lobelia cardinalis* hairy root cultures (HRCs) in response to the plant hormone jasmonic acid (JA) and a JA-loaded nanoparticle carrier (mesoporous silica nanoparticles (MSNPs)). Ultra-high performance liquid chromatography-mass spectrometry (UHPLC-MS) with high resolution mass spectrometry identified 12 *m*/*z* features only upregulated in the treatments with JA (free JA, JA-loaded MSNPs, and physical mixtures of JA and MSNPs) relative to a control experiment (without JA) or exposure to MSNPs alone. Putative identifications for these compounds elicited by JA included sesquiterpenoids (rishitin), monolignols (elemicin), and coumarins (7-hydroxycoumarin derivatives), respectively, and roughly correlated with the levels of JA that was measured in the respective HRCs. Signature *m*/*z* analytes associated with exposure to MNSPs were identified across treatment of bare MNSPs, JA-loaded MSNPs, and physical mixtures of JA and MSNPs and are consistent with a general stress response. The study demonstrates the ability to differentiate the elicitation effects of a biotic elicitor and its delivery system in untargeted elicitation studies, with a goal of designing nanocarrier systems for effective production of secondary metabolites.

## Abstract 2

The role of nanoparticle carriers of an exogenous plant hormone on elicitation, which is responsible for the chemical defense of plants in response to environmental stressors, is investigated from changes in production of secondary metabolites.

## Graphical abstract

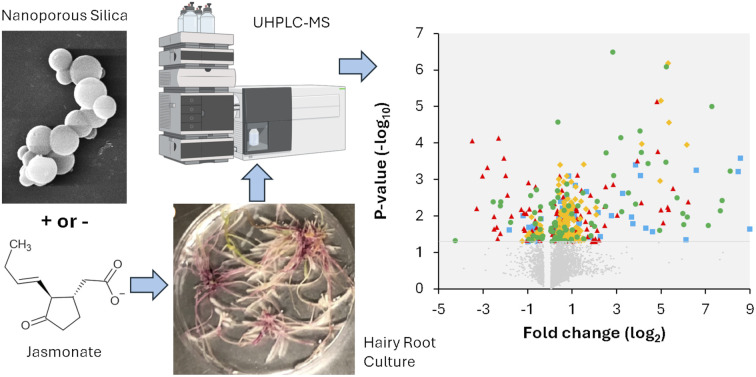

## Introduction

Plants produce a wide variety of organic compounds as a result of their diverse metabolic pathways. Primary metabolites are compounds essential for plant growth, development, and reproduction and include amino acids, nucleic acids, fatty acids, and carbohydrates. These biomolecules are structurally conserved across living organisms and are the building blocks for secondary metabolites. Secondary metabolites are not essential for plant physiology, but may confer survival advantage as chemical defenses in response to various environmental factors.^1,2^ Secondary metabolites are produced to combat any number of environmental stressors in the plant's natural environment. Elicitation of secondary metabolites involves intentionally evoking a metabolic stress response to learn about the effects of different stressors on plant metabolism^3–5^ and (perhaps, more importantly) to overproduce secondary metabolites with commercial potential.^6^

**Fig. 1:**
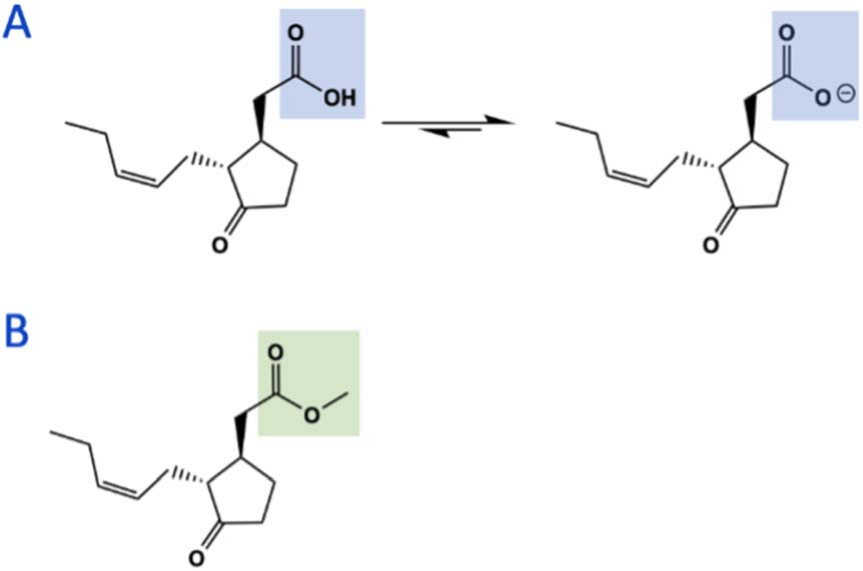
Structures of jasmonic acid (A) and methyl jasmonate (B) with their respective carboxylic acid and ester functional groups highlighted.

The desired stress response through elicitation can be achieved using controlled wavelengths of light,^7^ crude mycelial extract of the species *Fusarium* solani,^8^ and common additives such as cyclodextrin, magnesium chloride, and hydrogen peroxide.^9^ Stress hormones in plants, such as methyl jasmonate (MJ) and jasmonic acid (JA) (Fig. 1), are also widely studied elicitors.^8–16^ Since these compounds function to modulate metabolism in response to various environmental stressors,^17,18^ studying the metabolite changes that occur in a given plant species with exposure to the compounds provides valuable insights into how the plant handles stressful stimuli. Indeed, JA and MJ are well-established elicitors and have been shown to induce production of a wide variety of secondary metabolites including flavonoids,^19–21^ alkaloids,^19^ terpenoids,^22^ and phenylpropanoids.^20,21^

Engineering *in vitro* plant cultures, such as hairy root cultures (HRCs), for the production of metabolites on a larger scale addresses the unsustainability of harvesting low-concentration secondary metabolites from naturally grown plant material.^9,23^ Hairy roots are a pathological phenotype that occur naturally as the result of infection of plants with bacterium *Rhizobium rhizogenes*.^24^ This bacterium inserts a root-inducing plasmid into the plants genome, which thereby results in hairy root formation at the wound sites. HRCs can be readily propagated as independent explants, and are therefore a sustainable alternative to naturally grown plant material.^23^ Generally, HRCs also have the advantages of growing at a fast rate and may produce relatively higher levels of secondary metabolites compared with naturally grown plants. Production of bioactives such as shikonin and taxol have already been industrialized using elicitation strategies with HRCs.^19^

Elicitation requires careful consideration of both elicitor solubility in the media and cell membrane permeability to effectively deliver the elicitor through passive uptake.^5^ Delivery challenges for elicitors are illustrated by the model elicitors MJ and JA. Above its p*K*_a_ of 4.5,^25^ JA predominantly exists as the deprotonated carboxylate (Fig. 1A) in the cytosol of a plant cell, which is typically around 7.4.^26^ Active transport plays a role in the distribution of free anionic JA across the cell membranes.^27^ MJ is the methyl ester of JA, and it is uncharged. This reduces its aqueous solubility relative to JA,^28^ but allows passive transport across cell membranes. While JA may be more prevalent in the plant species, MJ is an active exogenous elicitor.^29^

Nanoparticles (NPs) address the challenges of delivering biotic elicitors for increased and prolonged production of targeted bioactive compounds in plant cultures.^30^ Nanoparticles can act as both direct replacements of biotic elicitors or as carriers for elicitors. Nanoparticle elicitors include metallics (*i.e.* silver, titanium oxide, zinc oxide, cobalt, or iron oxide NPs), bimetallics (*i.e.* iron and zinc NPs), magnetic, silica, and carbon-based NPs.^31,32^ While metallic NPs show increased elicitation of a wide range of bioactive compounds, their mechanism of action is often through a general increase in oxidative stress (rather than targeted elicitation) and research has shown high cytotoxicity associated with them.^33^

Biocompatible nanoparticles (*e.g.*, silica, chitosan and poly(lactic-*co*-glycolic-acid) nanoparticles) have been leveraged as carriers either through encapsulation or surface attachment of the biotic elicitor.^33,34^ Delivery of MJ by chitosan nanoparticles resulted in prolonged elicitation of flavonoids and phenolics in the species *Oryza sative* L. *Japonica* relative to free MJ in solution.^33^ This was due to decreased toxicity attributed to slow release from the nanoparticles.^33^ In another study with *Arabidopsis thaliana* the controlled release of salicylic acid (SA) from disulfide-functionalized mesoporous silica nanoparticles (MSNPs), which have a high loading capacity due to high surface and nanoporosity, resulted in sustained expression of plant defense gene PR-1 in comparison to free SA.^35^ Margaritopoulou *et al.* reported genetic activation of the JA pathway in *Arabidopsis* by JA delivery using chitosan nanoparticles.^36^ Employing nanomaterial elicitors with reduced cytotoxicity, particularly larger materials that are more readily recovered, also address concerns regarding post-elicitation disposal following the applications of the metabolites^33^

In addition to providing large surface areas and tunable morphologies (pore size and particle size),^37,38^ versatile functionalization strategies exist for MSNPs that can be used to increase nanoparticle uptake by cells,^39,40^ reduce cytotoxicity,^41^ and increase elicitor loading through affinity-based or electrostatic interactions.^41^ The driving force for the release of drugs from MSNPs is the diffusion of the drug from the pores, which can be tuned by modifying the nanoparticle surface with a chemical moiety that interacts more strongly or weakly with the desired cargo, *e.g.* cationic groups for JA.^42^ For example, Li *et al.* reported the development of functionalized MSNPs for delivery of salicylic acid and demonstrated extended release in glutathione-rich environments.^43^ The specificity that can be achieved in MSNPs has been used in cancer therapies to reduce side effects of toxic drugs by keeping them “contained” in the nanoparticle until the drug is eventually released into its local environment to carry out its desired therapeutic effect.^44,45^ The surface engineering of non-toxic MSNPs for the targeted harvesting of secondary metabolites from plant cell cultures has also been demonstrated.^46^

A challenge of evaluating the elicitation response of nanoparticle carrier systems is distinguishing the impact of the elicitation stress response induced by the uptake of the nanoparticles, which is independent of the response to the delivered biotic elicitor. While the biotic elicitor response may be targeted to a specific metabolic pathway and address a hypothesis, the stress response of nanoparticle uptake is untargeted and expected to impact a diverse range of metabolites, some of which may overlap with the effect of the biotic elicitor. For example, both phenolics^16,20,21,47,48^ and flavonoids^20,47–49^ are affected by jasmonic acid^16,20,21^ and a wide range of nanoparticles.^47–49^ Therefore, it is especially important to differentiate between which elicitor is causing the observed metabolite changes. This study employs mass spectrometry-based metabolomics techniques to distinguish between the metabolic response of the cells to biotic and nanoparticle stressors. Mass spectrometry is one of the most important analytical techniques used in the field of metabolomics due to its high sensitivity and ability to derive chemical formulae for unidentified metabolites when using a high-resolution mass instrument.^50^ In an untargeted metabolomics approach, all possible metabolites are identified and compared across samples relative to a control. Biological samples can easily contain hundreds to thousands of metabolites present in a wide range of concentrations and therefore make analysis extremely tedious,^51^ but the use of data mining software such as MZmine enables detection and deconvolution of large quantities of multidimensional data using computational methods.^52,53^

**Fig. 2:**
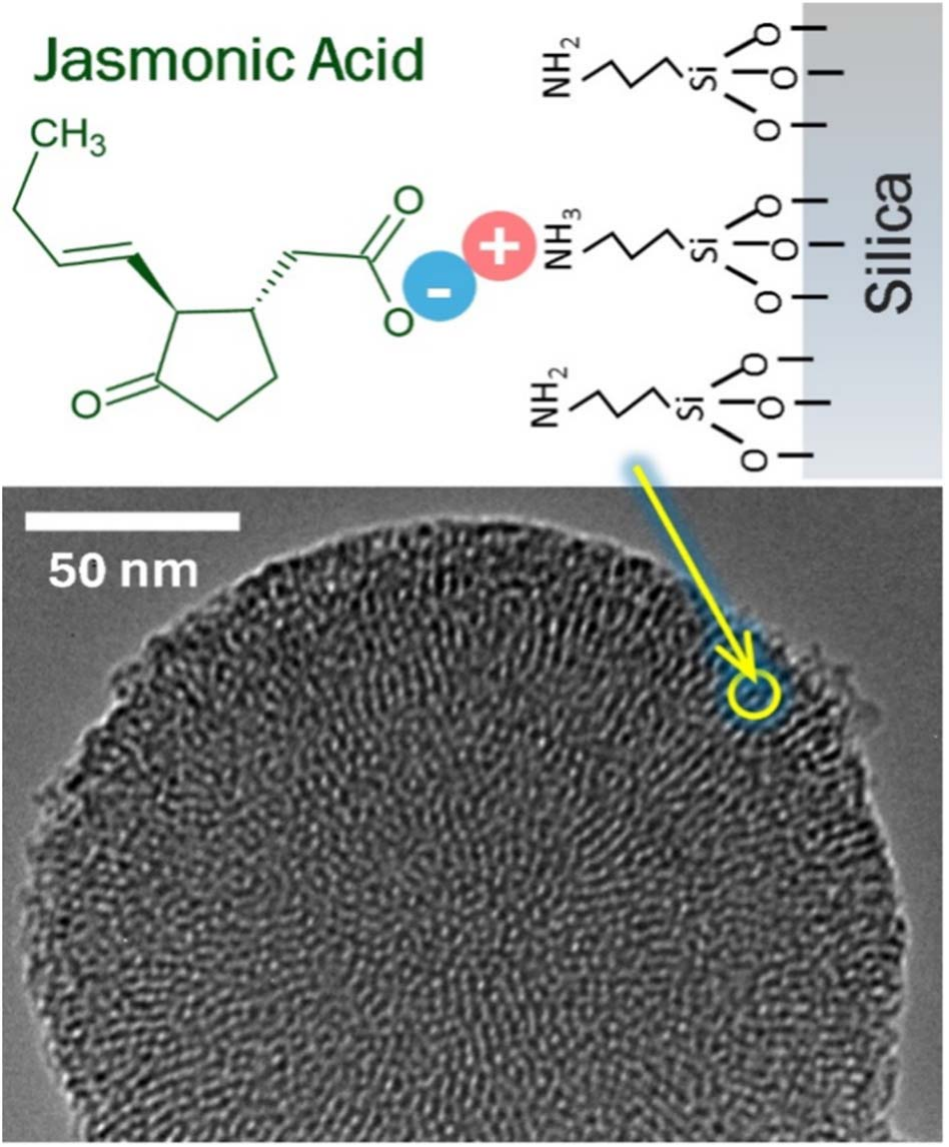
Schematic of the mechanism of electrostatic interaction-based loading of deprotonated jasmonic acid onto the protonated aminopropyl-modified surface of a mesoporous silica nanoparticle (MSNP). The transmission electron micrograph shows a representative MSNP with radially oriented pores prepared using the procedure discussed below.

In this study, the impact of delivering JA using mesoporous silica nanoparticles as the biotic elicitor carrier (see Fig. 2) on the untargeted production of secondary metabolites is evaluated using HRCs of *Lobelia cardinalis* as a model system. *L. cardinalis* is a plant species recognized for its medicinal properties^54–56^ and recently discovered to have anti-dopaminergic activity.^55,57^ To test the hypothesis that the effectiveness of MSNP delivery of biotic elicitors could be quantified and compared to the metabolic response of the biotic elicitor and the carrier, JA (negatively charged) was loaded on amine-functionalized MSNPs, whose positive surface charge not only promotes electrostatic JA loading (Fig. 2), but also is critical to promote the uptake into HRCs. We previously showed that amine-functionalized MSNPs are readily taken up and excreted by *L. cardinalis*.^46,58^ This is hypothesized to increase enhance JA intracellular transport to activate different metabolic pathways than the free acid.

*L. cardinalis* HRCs were exposed to free JA, unloaded MSNPs, a combined treatment of free JA in the presence of unloaded MSNPs, and MSNPs loaded with JA (JANP). Ethanolic extracts of roots from each treatment were analyzed by UHPLC-MS, and a broad range of mass features were identified. Metabolite profiles were evaluated to determine up- and down-regulated features with statistically different relative abundance compared to a control treatment with no JA or MSNP exposure. Features of secondary metabolites elicited by the biogenic elicitor JA and the nanoparticle carrier MSNPs were distinguished and the commonality of these features in the delivery of JA by nanoparticles was established.

## Materials and methods

### Materials

Ethanol was obtained from Alpha Aesar. Deionized (DI) water was acquired from Alpha Aesar, while Optima water used in UHPLC method was purchased from Fisher Scientific. Murashige and Skoog basal medium was obtained from Sigma-Aldrich, and jasmonic acid (≥98% pure) was purchased from Cayman Chemicals. The following were used for the synthesis, functionalization, and characterization of MSNPs: tetraethyl orthosilicate (TEOS, 99.9%, Thermo Scientific), cetyltrimethylammonium bromide (99.8%, MP Biomedicals), anhydrous ethanol (Koptec), ammonium hydroxide (ACS reagent, Supelco), 3-aminopropyl-triethoxysilane (APTES, 99%, Sigma-Aldrich), concentrated HCl (37%) (Sigma-Aldrich), and acid orange II (AO II, Thermo Scientific).

### Synthesis and characterization of mesoporous silica nanoparticles (MSNPs)

MSNPs were synthesized by controlled precipitation in the presence of a surfactant template using the procedure of Tan *et al.*^59^ to achieve accessible pores of approximately 2 nm in diameter. Briefly, cetyltrimethylammonium bromide (3.64 g), ammonium hydroxide (24.4 mL of 29.3 wt%), ethanol (112.7 mL), and DI water (70.9 mL) were combined in an Erlenmeyer flask and stirred. Tetraethyl orthosilicate (7.44 mL) was then added under constant stirring. The solution was aged at room temperature for 2 h. The precipitate was separated from solution *via* centrifugation, and the isolated particles were then washed three times with DI water. The particles were dried in an oven overnight at 100 °C. The surfactant template was then removed by placing the particles into an acidic wash of 150 mL dry ethanol and 3 mL concentrated HCl (aq) and stirring overnight. Surfactant-free particles were removed *via* centrifugation and washed with ethanol three times. Following the final washing steps, the particles were dried at 100 °C and transferred to a desiccator for storage.

Particles were amine-functionalized using a procedure adapted from Khan *et al.*^60^ Briefly, MSNPs (200 mg) were placed in an Erlenmeyer flask and heated in a 140 °C vacuum oven overnight. Ethanol (25 mL) was added to the particles and sonication was used to disperse the particles in the solution. To this stirring solution, 3-(aminopropyl)triethoxysilane (0.5 mL) was added dropwise under nitrogen. After being allowed to stir overnight at room temperature, the particles were separated *via* centrifugation, washed with dry ethanol (25 mL) three times, and subsequently placed in an oven to dry overnight at 100 °C. After one additional repetition of these washing and drying steps, the MSNPs with amine functionalization (MSNPAs) were transferred to a desiccator for storage.

The particle morphology was characterized using a Hitachi S-4300 Scanning Electron Microscope (SEM). Particles were dispersed onto a 15 mm aluminum stub using double sided carbon tape, excess materials were blown off with dry N_2_, and the samples were stored in a desiccator for 24 h. Prior to SEM analysis, the particles were coated with conductive Au–Pd alloy using an Emscope SC400 sputtering system. Average and standard deviation of particle diameters were calculated using 38 randomly selected particles using ImageJ Software. Surface characterization was performed using nitrogen adsorption conducted at −196 °C with a Micromeritics TriStar 3000 gas sorption instrument. MSNPs before and after amine functionalization were degassed at 135 °C for 4 h under flowing N_2_ gas before analysis. The specific surface area was estimated using the Brunauer, Emmett and Teller (BET) isotherm, and the pore size distribution and cumulative pore volume by the method of Barrett, Joyner and Halenda (BJH) using the adsorption branch of the isotherm.^61^

The amine density on MSNPAs (mol amine per mg MSNPA) was quantified by binding an anionic dye (AOII) to the amine-functionalized particles and quantifying the amount of dye released when the pH of the solution was increased, as adapted from Noel *et al.*^62^ In this spectroscopic assay, 2.5 mg of MSNPAs was added to 1 mL of a 0.5 mM AO II aqueous solution (adjusted to pH 3 with HCl). After sonication for 30 min and incubation at room temperature for 15 min, the MSNPAs were centrifuged and washed with 2 mL additional aqueous solution at pH 3, to remove unbound dye. The dye-loaded particles were then resuspended in 2 mL of aqueous solution (adjusted to pH 12 with NH_4_OH) by sonication for 30 min, followed by incubation at room temperature for 15 min. At pH 12, the amine functional groups become uncharged and release the dye. The particles were then removed from solution by centrifugation and the concentration of dye in the supernatant was analyzed. Solutions of released dye were analyzed at 495 nm using a UV-Vis spectrometer and the concentration of the dye solution was determined from a standard curve. The dye release and recovery steps were repeated several times until the dye removed was undetectable. The total amount of dye recovered in these steps was used to determine the amine density.

### JA loading and quantification in MSNPs

An evaporative loading technique was used to load MSNPAs with jasmonic acid (JA). A solution of JA in ethanol (200 µL, 8.4 mg mL^−1^) was added to 40 mg of MSNPAs in a 1.5 cm diameter glass vial. After ensuring all particles were submerged in the solution, the vial was heated on a hot plate for 45 min to 1 h at 60 °C until the solvent was fully evaporated.

Thermogravimetric analysis (TGA) was conducted using a TA Instruments SDT Q600 to determine the quantity of elicitor loaded into the jasmonic acid-loaded particles (JANP). Between 5–10 mg particles were used for each measurement. Unloaded MSNPAs (from the same batch) were used as a reference. JANP weight loss was measured from the TGA in a temperature range of 100–900 °C with a heating rate of 10 °C min^−1^ to ensure weight loss was not attributable to the evaporation of water out of the sample. The difference between the MSNPA reference and JA_NP was determined to be the weight percentage of JA loaded. Final loaded concentrations of JA in JA_NP samples were calculated based on this weight percentage.

### Cultivation and elicitation of *Lobelia cardinalis* hairy root cultures (HRCs)

The leaves of *L. cardinalis* seedlings were co-cultured with the *Rhizobium rhizogenes* strain R1000 carrying the pEarlyGate plant vector. Putative hairy roots were excised from the leaf explants and selected for on 20 µg mL^−1^ BASTA for at least 8 weeks. The hairy roots were maintained on solid ½ MS medium supplemented with 3% sucrose, 0.5 g L^−1^ MES, 8 g L^−1^ phytagar, pH 5.8 at 24 °C in 16 h photoperiod.

Mature *L. cardinalis* HRCs were excised with lengths varying from 3–6 cm. To minimize biological variation that can be present in different locations in the tissue culture, roots without branching were chosen. Excision was executed with one cut per root, with the root apex left intact. Excess incubation media was removed by dipping roots in deionized water and manually removing any remaining gel. Fifteen HRC samples were created (one untreated control and four treatment groups with three biological replicates per treatment) by placing 222 ± 9 mg of hairy root tissue in airtight Petri dishes in 10 mL of ½ Murashige and Skoog basal media (MSm) on a rotary shaker at ambient temperature for 24 hours. As outlined in Table 1, in addition to a control vector that was not exposed to JA or MSNPs, treatments included 100 µM JA, unloaded MSNPs (0.38 mg mL^−1^), a physical mixture of 100 µM JA with unloaded MSNPs (0.38 mg mL^−1^), and jasmonic acid-loaded particles (corresponding to 100 µM JA and 0.38 mg mL^−1^ MSNPs). Each 10 mL media sample contained 10 µL of ethanol, representing the ethanol solution used to introduce JA into the medium.

**Table 1:** Treatments and media components for each set of elicitation experiments using 222 ± 9 mg of hairy root tissue placed in 10 mL of ½ Murashige and Skoog basal media (MSm) at ambient temperature for 24 hours

Treatment (abbreviation)	Medium
Control (C)	17.1 mM ethanol (EtOH) in MSm
Free jasmonic acid (JA)	100 µM JA + 17.1 mM EtOH in MSm
Unloaded MSNPs (NP)	0.38 mg per mL MSPA + 17.1 mM EtOH in MSm
Physical mixture of free jasmonic acid and unloaded MSNPs (JA_NP)	100 µM JA + 0.38 mg per mL MSPA + 17.1 mM EtOH in MSm
Jasmonic acid loaded MSNPs (JANP)	JA-loaded MSNPs corresponding to 100 µM JA+ 0.38 mg per mL MSPA + 17.1 mM EtOH in MSm

### Sampling HRCs for analysis

Hairy root tissue was carefully removed from treatment media, rinsed 3× with deionized water, and dried using paper towels. The homogenization procedure was adapted from those previously described.^63–66^ Tissue was transferred directly into 2 mL microtubes with 2.4 mm metal beads and 1 mL of 80 : 20 ethanol : water, and samples were extracted using 45 s cycles in an Omni Bead Ruptor 4 until tissue was fully homogenized. Hairy root extracts were centrifuged, and a 1 : 40 dilution with Optima water afforded a sample for UHPLC-MS analysis.

### UHPLC-MS analysis of metabolites

A Shimadzu HPLC equipped with a Nexera X2 LC-30AD pump was coupled with a Thermo Scientific Q-Exactive ESI-Orbitrap mass spectrometer for analysis of HRC sample extracts. A Kromasil EternityXT-C18 column with 1.8 µm particle size and dimensions of 2.1 × 50 mm was utilized for analytical separation. With a flow rate of 0.375 mL min^−1^ and a binary solvent system with 0.1% formic acid in Optima water and 0.1% formic acid in Optima acetonitrile (ACN), chromatographic starting conditions were 5% ACN and held for 2.5 min. The system was then ramped to 95% ACN over 9.5 min and subsequently held for 4.0 min. The solvent composition was then reverted to starting conditions over 2.0 min and held for 2.0 min. MS data were collected in positive ion mode, with scan parameters consisting of a scan range of mass-to-charge ratio (hereby designated as *m*/*z*) 100.0–1000.0, a mass resolution of 140 000, 3 microscans per scan, and an AGC target of 3 × 10^6^. The heated electrospray ionization (HESI) source was set to a sheath gas flow rate of 15 L min^−1^, a spray voltage of 3.5 kilovolts, and a capillary temperature of 280 °C.

#### Data processing

LC-MS data was processed with Thermo Scientific Xcalibur 4.4.16.14 software. This data was then analyzed using MZmine 2.53 data mining software to identify “features,” or *m*/*z*'s associated with a particular retention time (RT). Mass features were detected between RT 0.00–12.01 min and the reported features were identified using the centroid mass detection algorithm with the noise level set to 9 × 10^4^. The ADAP chromatogram builder was then used to construct chromatograms of mass features (minimum group size 5, group intensity threshold 1 × 10^5^, minimum highest intensity 2 × 10^5^, and *m*/*z* tolerance of 0.001 or 5 parts per million (ppm)). This feature list was processed through chromatogram deconvolution using the baseline cutoff algorithm (*m*/*z* center calculation of median, minimum peak height 2 × 10^5^, peak duration 0–2 min, and baseline level 1 × 10^5^). Isotopes were identified using the Isotope Grouper with *m*/*z* tolerance of 0.001 or 5 ppm, and RT tolerance of 0.25 min. The features were limited to a maximum charge of 2 with the most intense ion as the representative ion. The Join Aligner was used to build an aligned feature list (*m*/*z* tolerance of 0.001 or 5 ppm, absolute RT tolerance of 0.25 min, weight of *m*/*z* and RT assigned as 75% and 25%, respectively) that showed all *m*/*z* data juxtaposed for the 6 samples (2 treatments × 3 replicates per treatment) in a manner that allowed for direct comparison. The Gap Filling feature was then used to fill in missing peaks (Same RT and *m*/*z*, *m*/*z* tolerance of 0.001 or 5 ppm). Features not present in all three analytical replicates were removed from the aligned feature list. Each remaining feature was evaluated using two tailed unpaired *t*-tests with equal variance assumed. Each feature having a *p*-value of <0.05 was selected for evaluation of area counts for treatments relative to control. Features for which the ratio was within 0.8–1.2 (defined as± 20% of a ratio of 1.0 representative of no significant change^67^) were assigned as the normal range of metabolomic variation and filtered out. The raw data for the features falling outside this range was probed using the Thermo Xcaliber Qual Browser software. *t*-Tests were performed on GraphPad Prism 9.5.1 to determine statistical significance between the data sets. Furthermore, putative identifications were based on exact mass data derived from the Kyoto Encyclopedia of Genes and Genomes (KEGG) database. Observed analyte *m*/*z* was required to fall within a *m*/*z* tolerance of 0.001 or 5 ppm for compound to be assigned an identification.

## Results and discussion

### Synthesis, characterization, and loading of MSNPs

SEM confirms that the synthesized MSNPs are discrete and spherical particles (Fig. S1). The particle size distribution was determined to be 440 ± 140 nm using ImageJ software. The size of the particles (>100 nm) is consistent with an elicitation mechanism *via* uptake through attachment to the cell wall material.^68^ as observed for chitosan nanoparticles carriers of MJ (≈300 nm)^33^ and multiwalled carbon nanotubes (MWCNT),^69^ which are approximately 5–15 nm in outer diameter but micron-scale in length.^70^ Uptake of submicron particles in plant roots has been shown to be size-independent in the 100–700 nm diameter range,^71,72^ and we have previously found MSNPAs in the 150–200 nm diameter range to concentrate near and between cell walls throughout root tissue.^58^ Through nitrogen adsorption, the BET specific surface area of the MSNP was determined to be 854.6 ± 10.6 m^2^ g^−1^, and 699.6 ± 8.3 m^2^ g^−1^ for MSNPA after amine functionalization. The pore volume was initially 0.839 cm^3^ g^−1^ for MSNP and decreased to 0.693 cm^3^ g^−1^ for MSNPA due to the reduction of pore diameter by the amines. The pore size distributions determined by the BJH method (Fig. S2 and S3) are narrowly distributed and follow Gaussian distributions with a mean 2.1 nm pore diameter and standard deviation of 0.2 nm for both MSNP and MSNPA.

After surface modification by APTES (Fig. S4), the amine density was determined using a quantitative assay with anionic dye acid orange II.^35^ Using the specific surface area of the MSNPA and the amount of bound dye, the amine density was determined to be 0.232 µmol m^−2^, corresponding to an amine surface coverage slightly less than 10% of a monolayer of amines throughout the surface of the pores (3.3 mmol nm^−2^ based on a silane area of 0.5 nm^2^).^73^ The amine density was used to determine the amount of JA that was loaded, assuming 1 : 1 amine binding at full capacity. This corresponds to 200 µL of a 8.4 mg per mL JA solution used for loading 40 mg of particles.

**Fig. 3:**
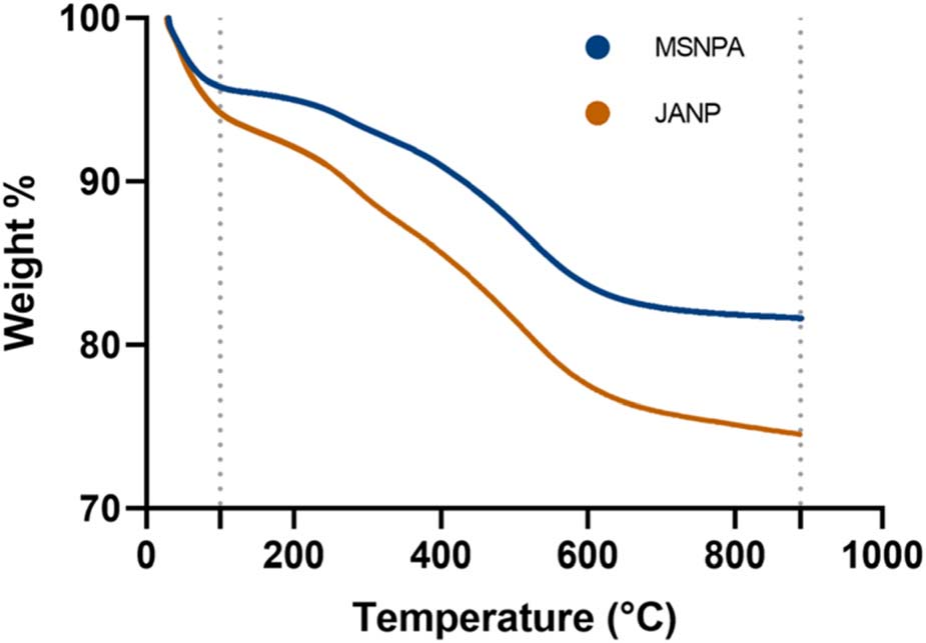
Weight change of JA-loaded MSNPAs relative to unloaded MSNPAs as determined by TGA analysis. Weight change was determined between the range of 100 °C and 900 °C, as annotated by the grey dotted vertical lines.

The particles used for loading included adsorbed water and aminopropyl groups, so the actual amount of JA loaded through evaporation on MSNPA was determined from the weight loss of the sample during TGA. TGA was performed for MSNPA with and without JA loading (Fig. 3). The initial weight loss below 100 °C is due to evaporation of residual solvent and water and is not included in JA loading calculations. From 100 °C to 900 °C, unloaded MSNPAs experienced a 14.2% decrease in weight. JANPs experienced a decrease of 19.7%, showing that JA contributed to 5.52 wt% of the loaded particles or 0.0552 mg JA per mg particle. This is 30% in excess of the amine-based capacity of the particles (0.042 mg per mg silica) but well below the amount of solid JA the pore volume could accommodate (0.73 mg mg^−1^).

### Cultivation and elicitation of *Lobelia cardinalis* HRCs

The treatments were designed to standardize the amount of JA and MSNPA incubated with the HRCs (Table 1). The medium for the HRC experiments with JA was prepared to achieve an elicitor concentration of 100 µM, a quantity that was determined from preliminary experiments where *L. cardinalis* HRCs were exposed to varying concentrations of JA for 24 hours and qualitatively examined over two weeks, showing that concentrations of JA higher than 100 µM were cytotoxic. Similar levels of JA or MJ have been used in previous elicitation experiments.^74^ From the TGA results quantifying JA loading on MSNPA, the concentration of JA-loaded NPs required to achieve 100 µM JA in the medium is 0.38 mg MSNP per mL. Because free JA was dissolved in ethanol to introduce it into the medium, all treatments also contain 17.1 mM ethanol. Representative *L. cardinalis* hairy root culture used in the elicitation experiments are imaged in Fig. S5. There were no noticeable differences in hairy root phenotype across each treatment over the course of exposure.

### UHPLC-MS analysis of metabolites

**Fig. 4:**
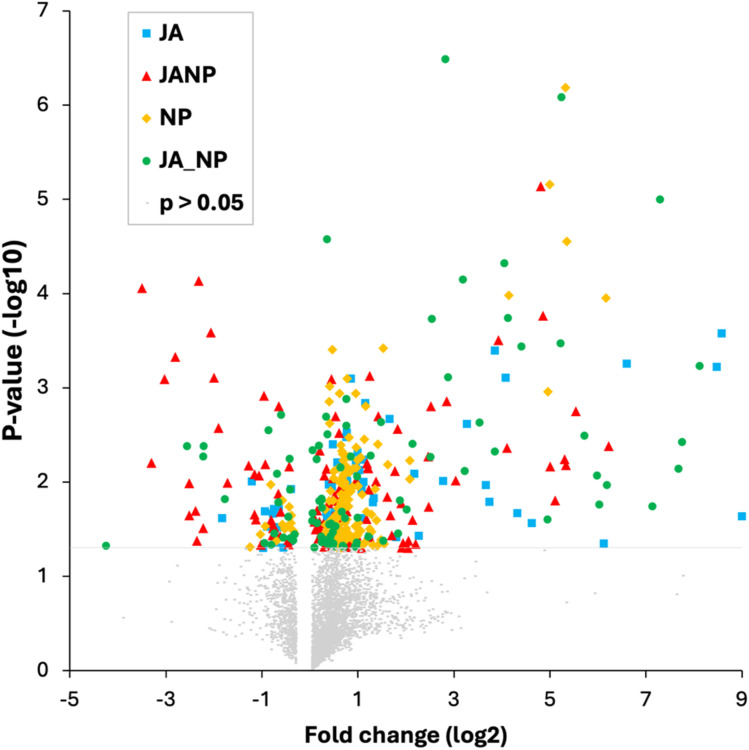
Volcano plot of all 1808 *m*/*z* features identified in the elicitation experiments across free JA, JA-loaded NPs (JANP), a physical mixture of JA and NPs (JA_NP), and unloaded NP samples by UHPLC-MS. The *x*-axis shows the magnitude of the fold change in each treatment control, where an arbitrary noise level of 1 × 10^3^ was used to calculate a ‘fold change’ value for features observed in an elicitation treatment but not the control. Features from 0.8–1.2× fold change assigned to be within the range of normal biological variation. The *y*-axis represents the *p*-value, with higher values indicating increasing statistical significance as a result of conversion to negative log values. The colored points represent features with *p*-values below 0.05 and deemed potentially significant. Blue squares represent features in JA elicitation, yellow diamonds represent features in NP elicitation, green circles represent features in JA_NP elicitation, and red triangles represent features in JANP elicitation.

A combined total of 1808 *m*/*z* features were identified from the homogenized HRC samples after exposure to control (untreated), JA (jasmonic acid solution only), NPs (nanoparticles only), JA_NP (a physical mixture of jasmonic acid solution and nanoparticles), and JANP (JA-loaded nanoparticles) treatments. The volcano plot in Fig. 4 summarizes the features found in the treatments and their level of significance according to the *p*-value and magnitude of their fold change relative to the untreated control. Features falling within the ratio of 80–120% abundance relative to control were excluded from the volcano plot as they were deemed within the limit of biological variation. Some features not detected in the control were present in the treatment samples. Abundance ratios for analytes undetected in controls were assigned an arbitrary noise level of 1000 to calculate a representative fold change value.

For the features described above, the KEGG database was used to assign putative identifications for 226 *m*/*z* features based on high resolution accurate mass data within a *m*/*z* tolerance of 0.001 or 5 ppm. Putative metabolites identified included a total of 50 putative alkaloids, five putative amide-type secondary metabolites, and four putative betalains across all 15 samples in this study. This finding is unsurprising since *Lobelia* species are known for producing alkaloids as their major secondary metabolites.^75^ Production of the nitrogenous compounds mentioned above requires their amino acid biosynthetic precursors; thereby, the identification of nine different amino acids in this study further supports the validity of the study. Additionally, a wide variety of putative phenolic compounds were identified, including 17 putative flavonoids, 16 putative monolignols, eight putative lignans, and 12 putative coumarins. This strongly suggested high metabolic flux through the phenylpropanoid pathways that have phenylalanine and tyrosine as biosynthetic precursors,^76^ which were two of the nine putative amino acids identified in the samples. This complete list of putative nitrogen-containing secondary metabolites, phenolics, and amino acids can be found in SI Table S2. It is also worth noting that a considerable number of putative terpenoids were also identified, but the number of possible identifications was much higher since terpenoid structures are highly diverse and many have the same chemical formula but many unique structural connectivities. We therefore limited the metabolite list to exclude putative terpenoid compounds.

Out of 1808 features identified across all the samples, 314 features were identified in which the fold change of the ratio was above 120% control or below 80% control for any of the treatments. Of these, only 167 had a *p*-value below 0.05 and were statistically significant. This feature list with full area count data is available in SI Table S1, along with any putative identifications assigned based on the KEGG compound library.

**Fig. 5:**
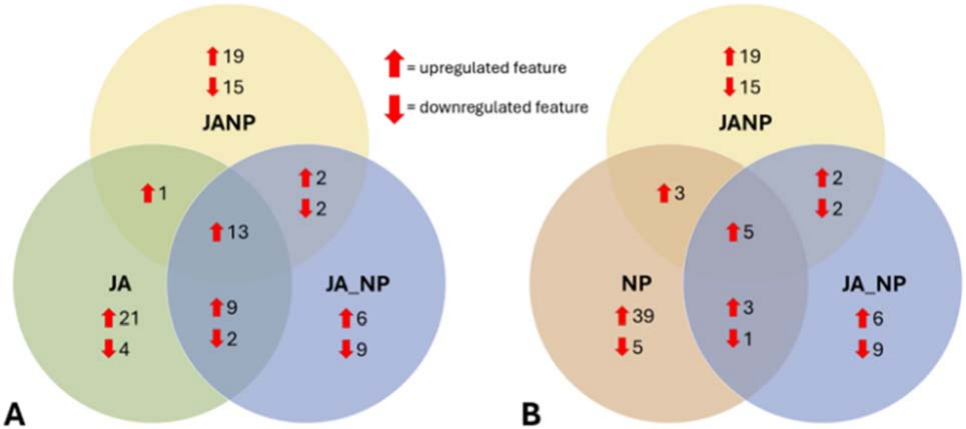
Venn diagrams summarizing overlapping *m*/*z* features identified in all elicitation experiments involving JA (A) and NPs (B) with significant changes, defined as features with area count ratios outside the range of 80–120% relative to control and a *p*-value below 0.05. Note: These diagrams include 159/167 features with the remaining eight features falling into minor categories not represented above.

The treatments associated with the 167 *m*/*z* statistically significant features (*p*-value < 0.05) are described in the Venn diagram in Fig. 5, which distinguishes between the three treatments with JA (Fig. 5a) and the three treatments with NPs (Fig. 5b). These observations of the elicitation response (upregulated and downregulated metabolites) and the individual contributions of JA and NP are described in more detail below.

### Effect of JA on secondary metabolites

**Fig. 6:**
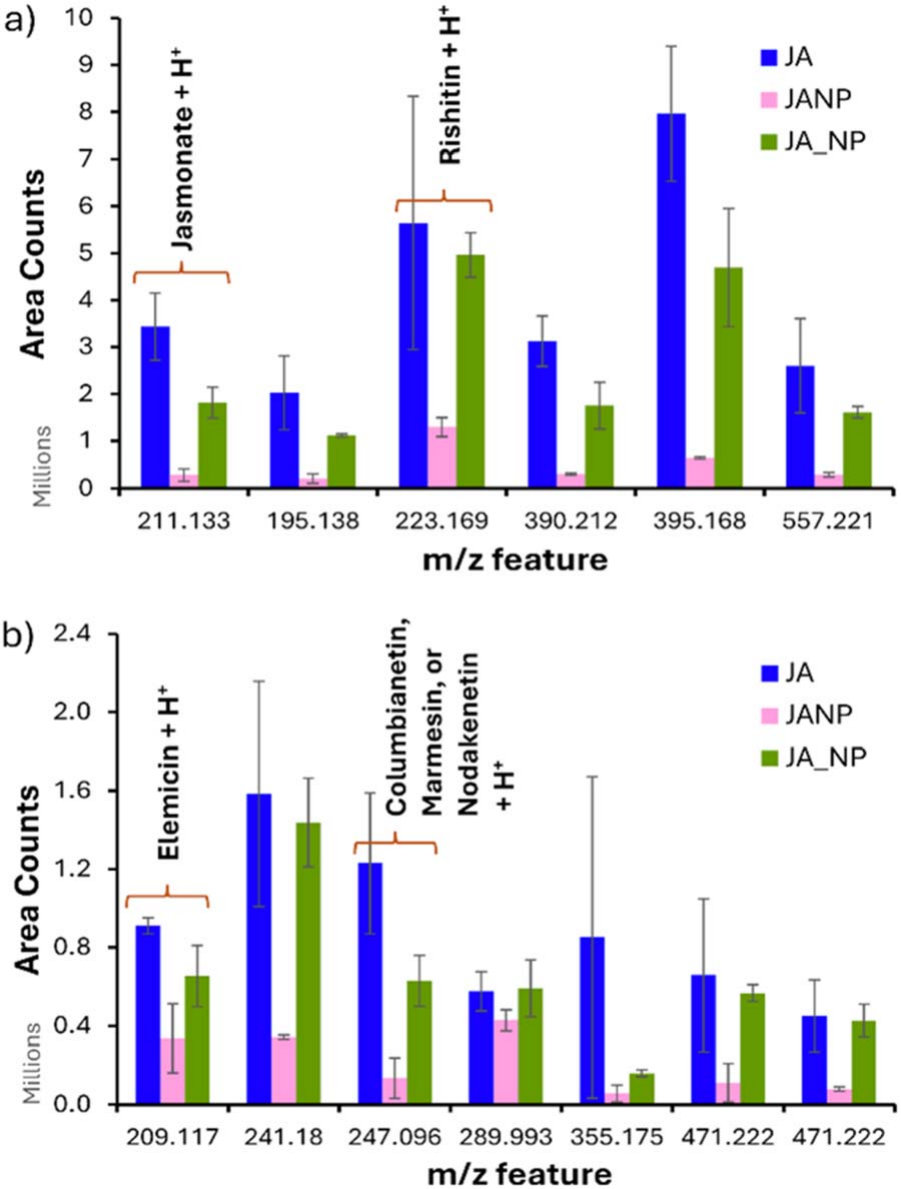
The *m*/*z* features identified in the elicitation experiments as upregulated as a result of JA elicitation. The data are presented in two figures (a and b) with different scales for clarity. Bars represent mean ± SD (*n* = 3 biological replicates). Elicitor jasmonic acid is distinguished with red brackets. The only *m*/*z* feature also present in the control is *m*/*z* 289.993 at an abundance of 2.73 × 10^5^ (see Table S1). The two *m*/*z* features at 471.22 (b) represent compounds with different retention times.

All three treatments in which the HRCs were exposed to JA (Fig. 5a; 13 overlapping features) resulted in detectable levels of JA as well as the elicitation of 12 additional *m*/*z* features that were not detectable in the control or the NP treatment. Only one of the 13 overlapping features present in all JA treatments, *m*/*z* 289.993, was also detectable in the control (abundance of 2.73 × 10^5^). We infer the delivery of the elicitor from the presence of JA (*m*/*z* 211.122 jasmonic acid + H^+^) in the HRCs (Table S1). JA is not detectable in the control (no JA exposure) but is present at ratios of 1.9 : 1 for JA : JA_NP and 12 : 1 for JA : JANP treatments (Fig. 6 (within red brackets)). The exposure of the HRCs to JA (free, NP-loaded, and a physical mixture of NPs and JA) were conducted at identical JA concentrations in solution, so the increased presence of JA in the extracts is attributed to its uptake. The treatment-dependent levels of JA in the plant cell extracts indicate that free JA effectively penetrates the hairy root tissues, resulting in the most abundance in the plant cell culture treatments. Of the JA treatments, the least amount of JA is observed for delivery of JA using nanoparticles, with the physical mixture of JA and nanoparticles resulting in intermediate levels of JA in the HRCs. As the amount of JA in the treatment increases, the abundance of the 12 *m*/*z* features that are a signature of JA elicitation (not detectable in the control) also increases (Fig. 6). In totality, this evidence is consistent with the measurement of the effects of exogenous JA elicitation on secondary metabolites.

A primary consideration for delivery of biotic elicitors directly to plant cell roots is the lipophilicity of the elicitor.^77^ The partition coefficient of a compound between octanol and water (*K*_ow_) is an established parameter to predict the uptake of a compound by a plant. Values of log *K*_ow_ between approximately 1 and 4 are consistent with uptake and mobility desirable for phytoremediation,^77^ and by extension, for elicitation in roots. Values of *K*_ow_ < 1 are associated with compounds that are too hydrophilic to pass through the cell membrane. At values of *K*_ow_ > 4, limited transport of the lipophobic compound may occur due to strong adsorption of the compound to the roots.^78^ The significant levels of JA uptake from solution are consistent with its log *K*_ow_ (estimated as 2.4)^79^ and its water solubility of 0.92 g L^−1^. The deprotonation of JA is expected to decrease its ability to permeate into the plant cells,^80^ but it is exactly this property that makes it amenable to delivery using amine-functionalized nanoparticles and therefore the focus of this study. The reduced level of JA observed in HRCs when exposed to physical mixtures of JA and NPs is consistent with reduced free JA in solution due to association with the positively charged MSNPs.

Since *L. cardinalis* produces primarily alkaloid species as their defensive metabolites,^81^ alkaloid compounds were hypothesized to be upregulated in response to jasmonic acid elicitation. In particular, upregulation of the major *L. cardinalis* compound lobinaline or one of its *N*-oxide derivatives was expected. Of the 12 signature features upregulated in response to jasmonic acid, however, only three were assigned putative identifications and surprisingly none of these were putative alkaloids. Rishitin (*m*/*z* 223.169), elemicin (*m*/*z* 209.117), and columbianetin/marmesin/nodakenetin (*m*/*z* 247.096) were among the putatively identified features and belong to the sesquiterpenoid, phenylpropanoid, and coumarin metabolite categories, respectively. These putative compounds are further described below. Importantly, these identifications are based solely on high resolution accurate mass data, and unambiguous confirmation of these analytes requires additional analyses such as comparison of retention times and tandem mass spectra with authentic standards.

The sesquiterpenoid rishitin is known for its antifungal properties as well as plant-growth retardant effects.^82^ Rishitin, as well as one of its glycoside derivatives, also have antibacterial activity.^83^ Therefore it is unsurprising that rishitin abundance increases in elicitation studies using bacterial and fungal extracts.^84,85^ Rishitin accumulation was also observed in elicitation studies with arachidonic acid and eicosapentaenoic acid,^86^ as well as several abiotic elicitors such as UV light and mercuric acetate.^87^ The abundances of the metabolite putatively identified as rishitin were 5.6 × 10^6^ ± 2.7 × 10^6^, 1.3 × 10^6^ ± 2.0 × 10^5^, and 5.0 × 10^6^ ± 4.7 × 10^5^ area counts for JA, JANP, and JA_NP, respectively (Fig. 6).

The monolignol-type compound elemicin was putatively identified in abundances of 9.1 × 10^5^ ± 4.0 × 10^4^, 3.4 × 10^5^ ± 1.8 × 10^5^, and 6.5 × 10^5^ ± 1.6 × 10^5^ area counts for JA, JANP, and JA_NP, respectively (Fig. 6). This compound was recently isolated from the essential oil of the ayurvedic medicinal plant nutmeg, *Myristica fragrans*, and determined to have antioxidant, antibacterial, and antifungal properties.^88^ Elemicin has been further recognized for its anti-acetylcholinesterase and antiviral activities.^89^

**Fig. 7:**
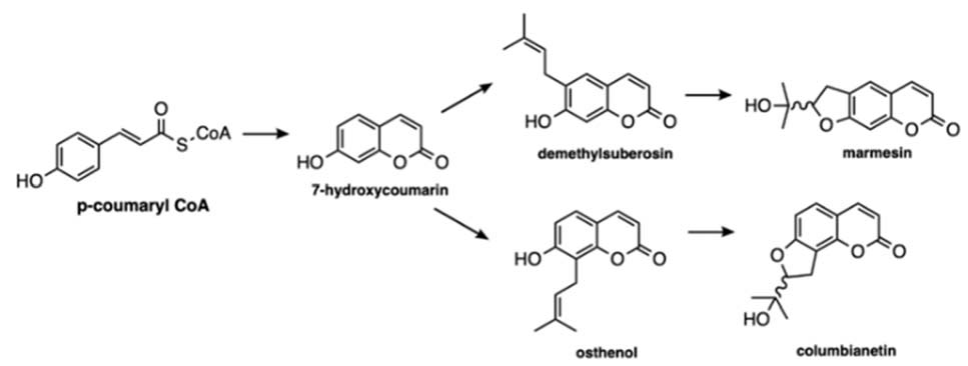
The biosynthetic pathway for columbianetin and marmesin synthesis from the precursor *p*-coumaryl coenzyme A.

The *m*/*z* 247.096 observed only in response to treatments exposing the HRCs to JA (Fig. 6), was identified in abundances of 1.2 × 10^6^ ± 3.6 × 10^5^, 1.3 × 10^5^ ± 1.0 × 10^5^, and 6.3 × 10^5^ ± 1.3 × 10^5^ in JA, JANP, and JA_NP treatments, respectively. The three possible identifications for this feature are columbianetin, marmesin, and nodakenetin as the (M + H^+^)^+^ ion. Interestingly, all three metabolites are biosynthetically related coumarin-type compounds. Two of these metabolites (columbianetin and marmesin) are derived from the common metabolite 7-hydroxycoumarin in the phenylpropanoid metabolic pathway.^90^ This biosynthetic pathway is summarized in Fig. 7. 7-Hydroxycoumarin is derived from *p*-coumaryl coenzyme A, which is a major node that leads to several other sub-classes of secondary metabolites such as stilbenoids and lignans.^76,91,92^ 7-Hydroxycoumarin is converted into demethylsuberosin, the metabolite from which marmesin is synthesized.^76^ Nodakenetin is not explicitly shown to be part of the same pathway, but is the enantiomeric form of marmesin and is highly likely to be synthesized in the same manner as marmesin or converted directly from marmesin. 7-Hydroxycoumarin can also be converted to a metabolite known as osthenol, a structural isomer of demethylsuberosin, and the biosynthetic precursor for columbianetin.^76^

Columbianetin is a bioactive component of *Angelica pubescens* Maxim. *f. biserrata* Shan et Yuan extract that has been used in traditional Chinese medicine to treat rheumatic disease,^93^ and shown to have anti-inflammatory effects on human mast cells.^94^ Other studies indicated antioxidant activity as well as cytotoxicity.^95,96^ Marmesin, in addition to being anti-inflammatory,^97^ was also reported to be hepatoprotective,^98^ anti-angiogenic,^99^ and UV-absorbent,^100^ in addition to a large number of other biological activities.^101–104^ Nodakenetin also displayed anti-inflammatory,^105,106^ antioxidant,^106^ and anti-cancer properties.^107^ Both columbianetin and marmesin were reported in a series of elicitation studies focused on furanocoumarin accumulation in poison hemlock plants.^108^ Meier *et al.* describe columbianetin accumulation induction in silver nitrate, copper(ii) sulfate, chitosan, and cellulase elicitations and marmesin induction in the chitosan and alginic acid accumulations.^108^

The remaining eight *m*/*z* features common to all JA elicitation experiments are unidentified based on the KEGG database, but the high-resolution mass spectrometry technique allows the chemical formulae to be narrowed. Table S3 provides one or more likely putative chemical formulae for these eight *m*/*z* features along with their corresponding mass error. Of note, 14 of 18 putative formulae contain nitrogen atoms and suggest the presence of alkaloids or other amino acid-related secondary metabolites. This is consistent with the hypothesis that alkaloids and their precursors are the main contributors to the biological activity of *L. cardinalis*. The remaining four putative formulae are composed of only carbon, hydrogen, and oxygen atoms and could fall into multiple metabolite categories such as terpenoids or flavonoids.

**Fig. 8:**
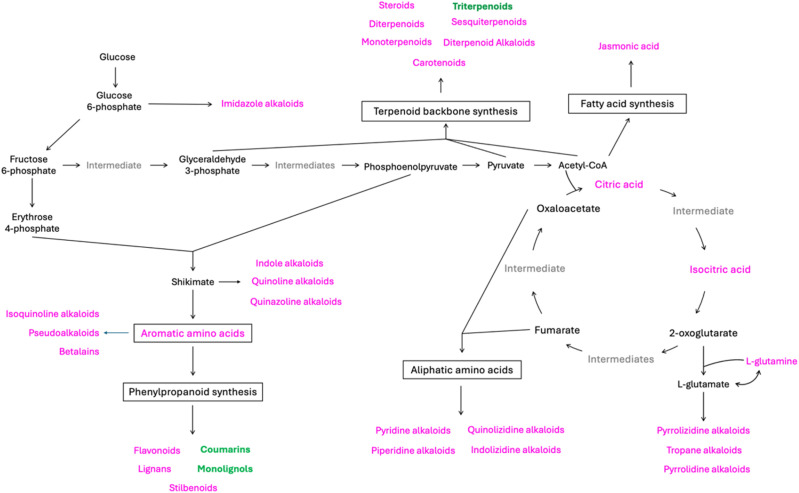
Representation of the classes of secondary metabolites observed in *L. cardinalis* hairy root cultures. The classes of compounds in pink are metabolites observed across the 15 samples (control and four treatment groups with three biological replicates) and consistent with the pathways shown in the figure. These metabolites are listed in Table S2. Jasmonic acid elicitation in *L. cardinalis* (observed in HRCs exposed to free JA, JA-loaded nanoparticles, and a physical mixture of free JA and nanoparticles) results in the upregulation of unique compounds in green, putatively identified as triterpenoids, monolignols, and coumarins.

Fig. 8 summarizes the overall effect of JA on *L. cardinalis* metabolism. While the exact compound classes the eight unidentified compounds have not been identified, putative chemical formulas for the unidentified compounds suggest that alkaloids or other nitrogenous metabolites likely play a large role in these metabolite changes. Further structural elucidation of these putative identifications using orthogonal approaches are critical for acquiring the full picture of the effect of JA on this system.

### Effect of NPs on secondary metabolites

**Fig. 9:**
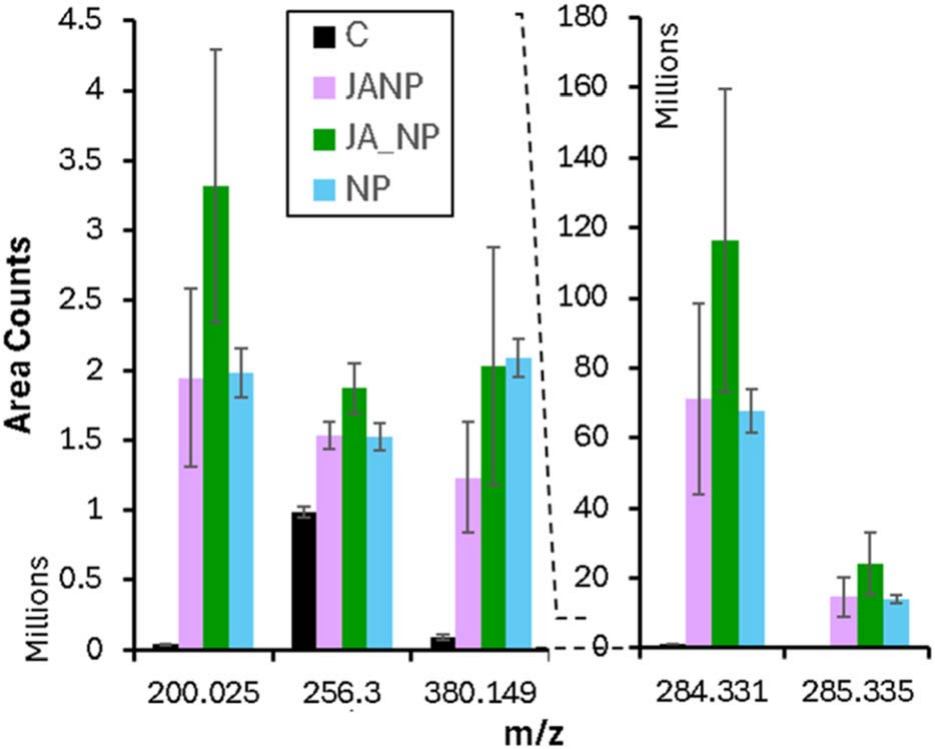
The *m*/*z* features identified in the elicitation experiments as upregulated following NP elicitation. Bars represent mean ± SD (*n* = 3 biological replicates).

The effect of the exposure to NPs on the secondary metabolites extracted from *L. cardinalis* HRCs was evaluated for all NP treatments (unloaded NPs, physical mixtures of NPs and JA, and JA-loaded NPs). Fig. 5B summarizes the features that were up- and down-regulated in the different treatments involving NPs. The common elicitation response to exposure to NPs observed across these treatments was the upregulation of five features shown in Fig. 9. Unlike elicitation due to the biotic elicitor JA, upregulated compounds associated with NPs were also present in the control.

While none of the five features identified above were assigned putative identifications, the exact mass data once again acquired allows us to obtain putative chemical formula for the analytes. As shown in Table S4, it is likely that *m*/*z* 200.025 is a putative oxygen-containing hydrocarbon compound, while *m*/*z* 256.191 and *m*/*z* 380.149 are likely putative alkaloids or other nitrogenous metabolites. Detailed mechanisms of nanoparticle elicitation have yet to be fully understood, but literature clearly shows that nanoparticles interact with cells and cause oxidative stress, so it is possible that these features described are produced as part of the oxidative stress response.^109^ This response involves the generation of reactive oxygen species (ROS), which triggers signaling cascades such as the mitogen-activated protein kinase (MAPK) that convey cellular distress and prompt the production of protective antioxidant metabolites.^109,110^ Other groups conducting elicitation studies have demonstrated the use of assays to measure oxidative stress biomarkers, such as hydrogen peroxide (H_2_O_2_) and malondialdehyde (MDA),^16,111^ and key antioxidant enzymes such as superoxide dismutase (SOD)^16,111,112^ and peroxidase (POD).^16,70,111,112^ The lack of overlapping features characteristic of JA exposure (Fig. 6) and NP exposure (Fig. 9) indicates that the stress response of HRCs to these elicitors is fundamentally different.

Similar to this study, the elicitation of phenolic acids in *Salvia verticillata* L., a medicinal plant model, and the oxidative stress response were compared for a biotic elicitor (MJ) and a nanomaterial elicitor (MWCNT).^69^ Oxidative stress was measured by H_2_O_2_ production and lipid peroxidation and correlated with the production of phenolic acid–base phytohormones (*e.g.*, abscisic acid, salicylic acid, rosmarinic acid, and JA).^69^ Both the oxidative stress response and the production of these potential therapeutic phenolic acids were greater for *Salvia verticillata L.* exposed to MWCNTs (whose dimensions suggest interaction through attachment to the cell wall material) than MJ.^69^ Thus, nanomaterials (MWCNTs) were more effective than biotic elicitor in stimulating the production of in *S. verticillata*, confirming the importance of investigations that independently evaluate the role of biotic and nanomaterial elicitors in medicinal plant models.

### Effect of JA-loaded NP carriers (JANP)

Some *m*/*z* features changed in abundance only in the JANP treatment and suggest that the combination of jasmonic acid and nanoparticles may elicit a unique response when presented to the cells as a combined carrier-cargo system. This is consistent with the hypothesis that MSNPAs loaded with electrostatically bound JA are able to penetrate plant cells to moderate different metabolic pathways than free JA. There is a small (30%) excess of loaded JA beyond the electrostatic capacity of the MSNPA, but not enough to mimic the effects of the JA control. Indeed, the reduced level of jasmonate found in JANP samples (Fig. 6a) shows that its effect is not simply due to being released into the culture medium. Nineteen upregulated features and 15 downregulated features were significantly changed only in the JANP treatment (Fig. 5).

Among the 19 upregulated features, three putative metabolites identified were buchananine, braxin C, and chaparrin. Buchananine has immunostimulatory activity,^113^ anti-fungal activity,^114,115^ and antibacterial activity.^114,116^ Braxin C, also known as ptaquiloside,^76^ was putatively identified in the JANP treatment and upregulated by 140% relative to the control treatment. It is a phytotoxin found naturally in the bracken fern plant, and known to cause cancer in livestock.^117^ Triterpenoid chaparrin, which was upregulated by 160% relative to control, displays some degree of antimalarial activity and cytotoxicity,^118–121^ but most literature suggests that this compound is more useful as a synthetic precursor for other bioactive compounds.^118–121^ While the plant's metabolic resources are being channeled towards the overproduction of these compounds likely to provide some kind of protective effect, the plant's resources are simultaneously being diverted away from the pathways producing the 15 uniquely downregulated metabolites, including putatively identified entadamide A and miraxanthin I.

The unique response to JANPs represents an interesting potential avenue of research in the area of nanoparticle-assisted elicitation. Furthermore, time-dependent elicitation studies may reveal the potential for longer term release using JANPs. Longitudinal studies of elicitation behavior could be further supported by investigations of JA release from nanoparticles as a function of nanocarrier loading and pH. Since this study was intentionally designed to compare the same levels of JA in solution and in carrier systems, it is therefore limited by the aqueous solubility of JA. JANPs could be utilized in future investigations to introduce JA at higher concentrations into HRCs to investigate the hypothesis that time and concentration could magnify the effect of elicitation using JANPs.

An investigation of the elicitation of plant cell cultures by MJ-loaded polymeric chitosan nanoparticles relative to empty polymeric chitosan nanoparticles (>200 nm diameter) highlights the need to address solubility when designing for pro-longed elicitation effects^33^ Both MJ-loaded chitosan NPs (CNPs) and empty CNPs significantly enhance the production of phenolics in model rice cell suspension cultures throughout a period of 28 days, with the greater elicitation response from the MJ-loaded chitosan NPs. An enhancement in flavonoid production, a more complex biosynthetic stress response than phenolic acid production, was not observed. The long-term elicitation effects were attributed to the gradual erosion of the chitosan polymer to release chitosan and MJ, with MJ having the key role in elicitation. Enhancing the encapsulation of the poorly water soluble MJ during chitosan nanoparticle synthesis is suggested as a critical step to increasing the effectiveness of this nanocarrier system.^33^

## Conclusions

This study evaluated the effects of the elicitor jasmonic acid for *L. cardinalis* and differentiated the effects of utilizing nanoparticles as carriers for jasmonic acid for untargeted elicitation. A total of 1808 metabolite *m*/*z* features were identified using HPLC-MS analysis of which 167 features were statistically significant relative to the control. Jasmonic acid promoted the synthesis of 12 metabolites. Putative identifications among these are the sesquiterpenoid rishitin, the monolignol-type compound elemicin, and a putative 7-hydroxycoumarin derivative. This signature set of features was detected when jasmonic acid was present in the bulk solution, as well as in the presence of cargo-loaded nanoparticles. The abundance of these features correlated with the amount of JA. Thus, NP functionalization methods that improve the loading and delivery of the biotic elicitor (such as amine functionalization examined in this study) are critical to the design of nanomaterial carriers.

In exploring the elicitation of a wide variety of secondary metabolites in *L. cardinalis*, we were able to show the ability to distinguish the elicitation effects of jasmonic acid from those of the nanoparticles. In the case of features upregulated by JA, loading the elicitor into MSNPA (JANP) reduced the response over the period study, consistent with the association with the carrier leading to delayed release of JA into the cells. This supports the use of nanoparticle carriers to provide sustained release of elicitors in plant organ cultures. Nanoparticle-assisted elicitation can be further optimized for a particular elicitor by modifying the nanoparticle properties. For example, functionalization of the nanoparticle surface with different functional groups could allow for stronger or weaker interactions depending on the desired effect for the system. Here, electrostatic interactions were used to bind a sparingly soluble elicitor, which we hypothesize to lead to delayed release. Amine-functionalized particles had the additional benefit of promoting particle uptake by HRCs, and the mechanism of delivery can be further explored using pH-dependent release studies from MSNPA,^122^ with unfunctionalized MSNP serving as a control of the effect of simple pore diffusion without electrostatic attraction. Future studies can further tune the release profile, for example by adjusting pore size or by selection of the amine functional group with a p*K*_a_ close to the pH inside of the cell walls, as has been demonstrated for tumor cell delivery.^123^

Materials development has been an important focus of nanoparticle carriers for phytohormones, and the sustained release from MSNPA of anionic molecules such as JA in biological media is well known.^122^ Fewer studies have directly investigated the effect of delayed release in the response of plant cell cultures. In the present study, delayed release is inferred from the presence of JA and the correlation with corresponding levels of JA-associated metabolites in the HRCs. However, delayed release of JA does not fully explain the changes in metabolic profile when comparing metabolites induced by free JA and MSNP-loaded JA. An elicitation response is observed in JA-loaded nanoparticles (Fig. 5, where there are 19 upregulated and 15 downregulated features only for the JANP sample) that is different than JA, NP, or JA loaded with NP (JA_NP). The results are significant in light of the changes in phenotype observed by Margaritopoulou when *B. cinerea* was exposed to JA-loaded chitosan nanoparticles.^36^ Future experiments that directly measure the uptake of exogenous JA in HRCs would help to understand the role of transport (from active transport of free JA to passive transport in JA-loaded nanoparticles) on changes in the metabolic profiles.

This system was able to illustrate proof-of-concept for using nanoparticles as a delivery system for elicitors and differentiating between elicitation effects of nanoparticles and the elicitor. The application of nanoparticle carriers would be particularly useful, however, for elicitors with limited water solubility, such as methyl jasmonate.^124^ Essentially treating the elicitor as cargo within the nanocarrier system would allow for delivery of the elicitor in aqueous media despite low water solubility, which could widen the range of potential elicitation agents. Further tuning the functionality and size of the MSNP would allow for cellular or organelle targeting,^125^ and coupling of elicitation with nanoharvesting to enhance secondary metabolite production and separation in plant organ culture.

## Author contributions

Rachel Sutherland: methodology, validation, formal analysis, investigation, writing – original draft; McKenna Clinch: investigation, writing – original draft; Kristen Bruce: validation, investigation; Trent Rogers: methodology, writing – reviewing and editing; John Littleton: conceptualization, methodology; Bert Lynn: conceptualization, methodology, writing – reviewing and editing; Stephen Rankin: conceptualization, methodology, writing – reviewing and editing, visualization; Barbara Knutson: conceptualization, methodology, writing – reviewing and editing.

## Conflicts of interest

There are no conflicts of interest to declare.

## Supplementary Material

RA-016-D6RA03646E-s001

## Data Availability

The data supporting this article have been included as part of the supplementary information (SI). Supplementary information: characterization of MSNP particle size and pore size, representative image of *L. cardinalis* HRCs, *m*/*z* features with area counts statistically different than the control as a function of treatment, putative plant metabolites identified across all samples in the elicitation experiments, putative identification of metabolites upregulated due to jasmonic acid and also due to nanoparticle elicitation. See DOI: https://doi.org/10.1039/d6ra03646e.
