# Supplementary material for: Jasmonic acid and nanoparticle elicitation of L. cardinalis – differentiating elicitor contributions in nanoparticle delivery strategies
Source: RSC Adv. 2026 Jul 2;16(34):31930–43. doi: 10.1039/d6ra03646e (PMC13325022; doi:10.1039/d6ra03646e)
Supplement: RA-016-D6RA03646E-s001 [file RA-016-D6RA03646E-s001.pdf]

Supplementary Information (SI) for RSC Advances.
This journal is © The Royal Society of Chemistry 2026

Supporting Information

Jasmonic Acid and Nanoparticle Elicitation of *L. Cardinalis* – Differentiating Elicitor Contributions in Nanoparticle Delivery Strategies

Rachel P. Sutherland,[1] McKenna Clinch, [2] Kristen Bruce,[3] D. Trent Rogers, [3] John Littleton, [3] Bert C. Lynn, [1] Stephen E. Rankin[2,*] and Barbara L. Knutson[2,*]

[1] University of Kentucky, Department of Chemistry, 125 Chemistry/Physics Building, Lexington, KY, USA

[2] University of Kentucky, Department of Chemical and Materials Engineering, 177 F.P. Anderson Tower, Lexington, KY, USA

[3] Naprogenix™, UK-ASTeCC 145 Graham Avenue, Lexington, KY, USA

Corresponding Authors E-mails: stephen.rankin@uky.edu (S.E.R) and bknut2@uky.edu (B.L.K)

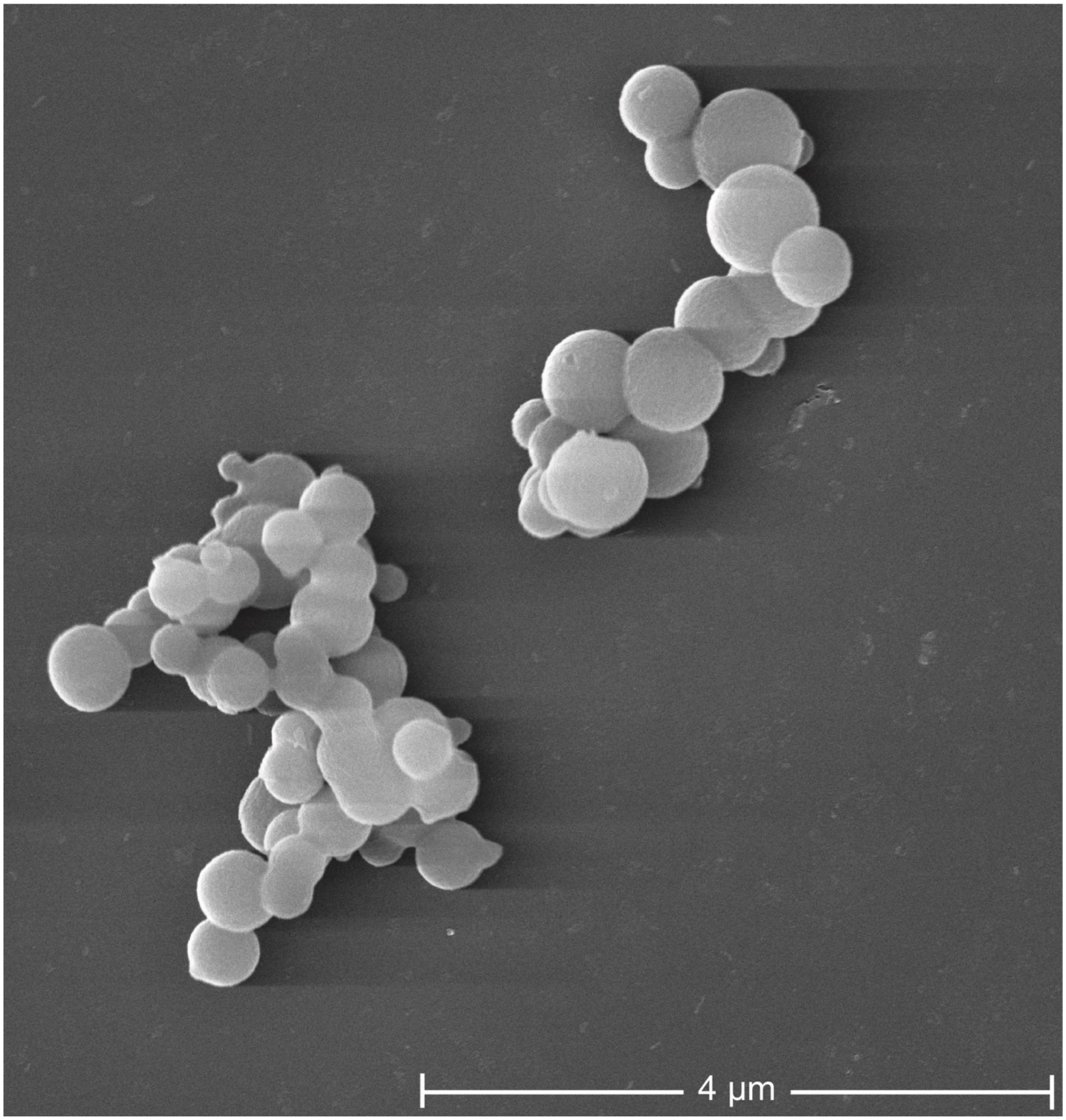

Fig. S1. SEM image of MSNP. From SEM images, the diameter of the MSNPs was determined to be 440 ± 140 nm using ImageJ software

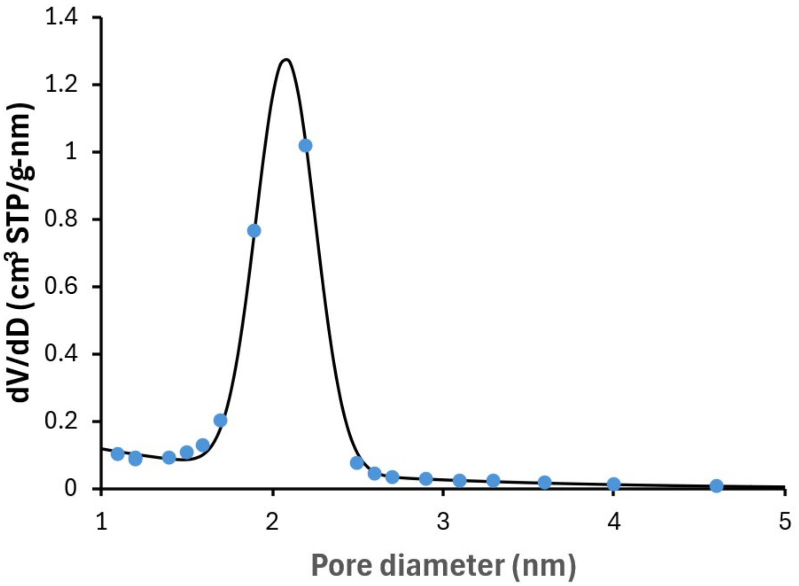

Fig. S2. Pore size distribution of MSNP determined from nitrogen adsorption using the BJH method using the adsorption branch. A Gaussian with an exponential baseline is fit to the measured data by nonlinear regression, giving a pore diameter of 2.1 ± 0.2 nm.

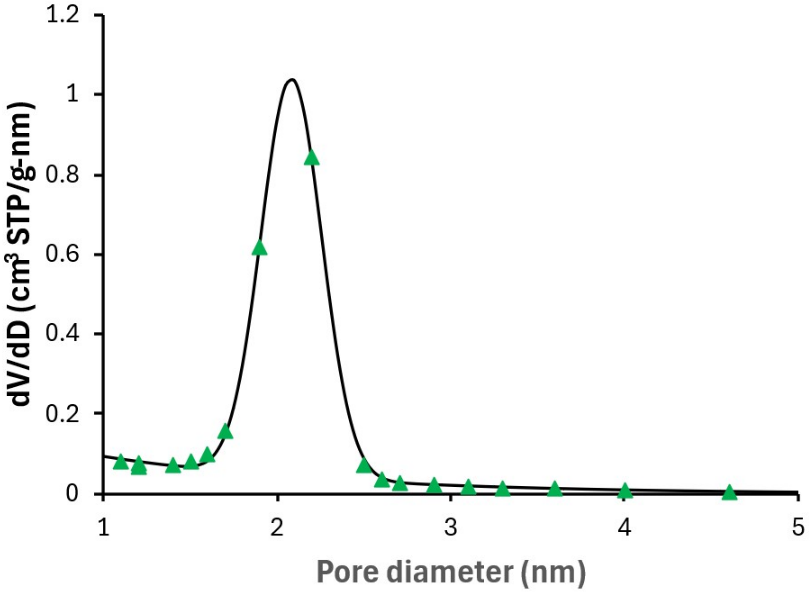

Fig. S3. Pore size distribution of MSNPA determined from nitrogen adsorption using the BJH method using the adsorption branch. A Gaussian with an exponential baseline is fit to the measured data by nonlinear regression, giving a pore diameter of 2.1 ± 0.2 nm.

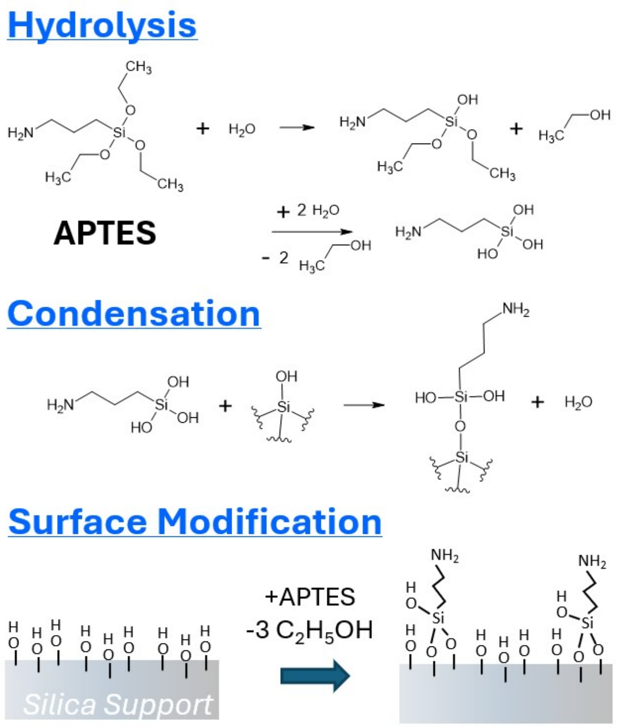

Fig. S4. Reactions of aminopropyltriethoxysilane (APTES) that lead to silica surface modification. *Hydrolysis* of ethoxyl groups leads to the formation of one or more hydroxyl groups, and *condensation* between the silane and hydroxyls on the silica surface lead to attached aminopropylsilyl groups. There can be between one and three siloxane bond attachments, but two is thought to be most common.

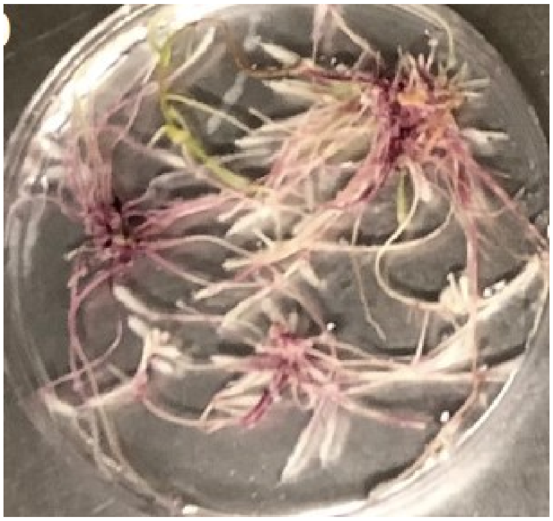

Fig. S5. Representative *L. cardinalis* hairy root culture used in the elicitation experiments. There were no noticeable differences in hairy root phenotype across each treatment over the course of exposure.

Table S1. The m/z features, putative identifications (if applicable), retention times, and average area counts of analytes identified in the elicitation experiments with statistically significant differences between treatment and control (C-control, JA-jasmonic acid only, JANP-JA-loaded nanoparticles, JA_NP- a physical mixture of jasmonic acid solution and nanoparticles NP- nanoparticles only) . Analytes identified in the elicitation experiments as upregulated as a result of JA elicitation (upregulated in in JA, JANP, and JA_NP treatments) are highlighted in green. Analytes identified in the elicitation experiments as upregulated in treatments involving nanoparticles are highlighted in blue. Analytes identified in the elicitation experiments as upregulated only in JANPs are highlighted in pink, while analytes downregulated in JANPs are highlighted in orange. An annotation of 'ND' indicates that metabolite was undetected in the sample.

Putative ID	m/z	RT (min)	C	JA	JANP	JA_NP	NP
(methionine +H)$^+$	150.058	0.5	4.17E+05 ± 9.00E+04	9.91E+05 ± 2.23E+05	1.24E+06 ± 8.14E+05	1.05E+06 ± 6.64E+05	6.23E+05 ± 1.34E+05
	150.076	0.5	8.14E+05 ± 3.92E+04	1.39E+06 ± 2.30E+05	1.39E+06 ± 6.56E+05	1.43E+06 ± 5.25E+05	1.18E+06 ± 3.39E+05
(entadamide A +H)$^+$	162.058	0.5	4.47E+06 ± 1.17E+06	4.26E+06 ± 7.14E+05	5.28E+05 ± 2.91E+05	3.91E+06 ± 8.92E+05	7.34E+06 ± 3.61E+06
	164.074	0.3	7.41E+05 ± 1.60E+05	1.75E+06 ± 6.41E+05	2.44E+06 ± 6.64E+05	1.76E+06 ± 1.44E+06	7.94E+05 ± 3.80E+06
(glutamine +Na)$^+$	169.058	0.3	1.87E+07 ± 1.18E+06	1.94E+07 ± 2.33E+06	1.36E+07 ± 2.42E+06	1.74E+07 ± 6.32E+06	1.48E+07 ± 7.97E+06
(tyrosine +H)$^+$	182.081	0.6	3.94E+06 ± 1.21E+06	7.16E+06 ± 1.53E+06	4.40E+06 ± 1.29E+06	8.34E+06 ± 3.81E+06	5.15E+06 ± 5.72E+06
	182.154	1.8	1.38E+06 ± 2.81E+05	8.12E+05 ± 1.15E+05	1.07E+06 ± 5.08E+05	7.92E+05 ± 3.67E+04	9.60E+05 ± 2.33E+06
	186.128	4.65	6.10E+05 ± 8.49E+04	6.80E+05 ± 3.96E+05	1.26E+06 ± 6.29E+05	8.17E+05 ± 7.52E+04	1.03E+06 ± 3.72E+05
	188.165	0.7	1.72E+06 ± 1.11E+05	2.42E+06 ± 1.14E+06	3.30E+06 ± 1.83E+06	2.42E+06 ± 1.36E+06	3.18E+06 ± 1.25E+06
	189.135	0.3	2.07E+06 ± 2.08E+05	2.73E+06 ± 1.07E+06	2.68E+06 ± 3.74E+05	2.54E+06 ± 1.29E+06	2.78E+06 ± 4.19E+05
	192.087	0.5	5.52E+05 ± 5.29E+04	1.04E+06 ± 3.80E+05	1.15E+06 ± 5.16E+05	9.34E+05 ± 5.94E+05	9.84E+05 ± 9.94E+05
	193.136	4.5	2.30E+06 ± 2.95E+05	2.36E+06 ± 1.39E+06	3.93E+06 ± 2.27E+06	4.92E+06 ± 2.50E+06	4.45E+06 ± 2.01E+06
	195.138	6.2	ND	2.03E+06 ± 7.86E+05	2.10E+05 ± 1.04E+05	1.12E+06 ± 3.62E+04	ND
	200.025	9.8	4.16E+04 ± 2.73E+03	4.37E+04 ± 1.25E+04	1.95E+06 ± 6.36E+05	3.32E+06 ± 9.77E+05	1.98E+06 ± 1.09E+06
	201.127	6.8	2.72E+05 ± 5.63E+04	4.63E+05 ± 5.87E+04	1.06E+06 ± 5.63E+05	4.12E+05 ± 1.61E+05	2.24E+05 ± 9.70E+05
	201.127	6	1.32E+05 ± 5.76E+04	2.55E+05 ± 1.64E+05	2.83E+05 ± 7.41E+04	2.48E+05 ± 1.50E+05	1.73E+05 ± 6.56E+04
	201.127	7.1	2.99E+05 ± 3.34E+04	4.88E+05 ± 7.51E+04	5.79E+05 ± 1.44E+05	4.18E+05 ± 7.47E+04	2.51E+05 ± 5.76E+04
	201.130	4.6	1.91E+05 ± 8.58E+04	3.19E+05 ± 1.81E+05	5.94E+05 ± 4.80E+05	3.17E+05 ± 7.48E+04	4.16E+05 ± 1.01E+05
	202.141	4.3	5.05E+05 ± 7.69E+04	6.69E+05 ± 3.38E+05	1.27E+06 ± 7.66E+05	6.99E+05 ± 1.18E+04	1.07E+06 ± 3.88E+05
	203.049	0.7	2.46E+06 ± 3.12E+05	2.66E+06 ± 3.42E+05	1.23E+06 ± 2.70E+05	2.56E+06 ± 3.82E+05	2.80E+06 ± 1.15E+06
	204.138	10.4	2.69E+06	2.36E+06	2.02E+06	2.34E+06	1.87E+06

			± 3.35E+05	± 2.18E+05	± 4.26E+05	± 8.53E+05	± 8.31E+05
	206.048	0.9	2.52E+06 ± 1.43E+05	3.19E+06 ± 3.65E+05	2.86E+06 ± 3.88E+05	3.27E+06 ± 3.82E+05	2.61E+06 ± 4.78E+05
(elemicin +H)+	209.117	5.4	ND	9.11E+05 ± 4.04E+04	3.37E+05 ± 1.76E+05	6.54E+05 ± 1.56E+05	ND
(jasmonate +H)+	211.133	6.3	ND	3.43E+06 ± 7.16E+05	2.85E+05 ± 1.35E+05	1.82E+06 ± 3.29E+05	ND
	212.852	0.3	2.17E+05 ± 2.85E+04	3.80E+05 ± 1.94E+05	5.46E+05 ± 4.53E+05	3.31E+05 ± 8.07E+04	5.39E+05 ± 3.20E+05
(citric acid/ isocitric acid +Na)+	215.016	0.5	2.03E+06 ± 3.37E+05	2.69E+06 ± 1.74E+06	7.15E+06 ± 2.91E+06	5.89E+06 ± 6.11E+06	6.92E+06 ± 5.41E+06
	219.098	0.4	2.83E+06 ± 5.51E+05	4.37E+06 ± 6.09E+05	3.26E+06 ± 7.40E+05	4.54E+06 ± 2.56E+06	3.74E+06 ± 4.51E+06
	219.138	6.3	8.04E+06 ± 5.37E+05	9.59E+06 ± 2.24E+06	1.15E+07 ± 9.45E+05	9.73E+06 ± 1.67E+06	8.09E+06 ± 2.51E+06
(rishitin +H)+	223.169	6.7	ND	5.64E+06 ± 2.69E+06	1.30E+06 ± 2.03E+05	4.96E+06 ± 4.74E+05	ND
	227.128	6.1	5.48E+05 ± 2.18E+04	7.09E+05 ± 4.53E+04	5.80E+05 ± 3.14E+04	6.19E+05 ± 5.03E+04	5.45E+05 ± 2.99E+05
	228.030	0.9	7.54E+05 ± 3.93E+04	9.67E+05 ± 9.27E+04	8.91E+05 ± 1.59E+05	1.02E+06 ± 1.16E+05	7.92E+05 ± 1.49E+05
	230.586	6.8	7.71E+04 ± 5.53E+03	1.76E+05 ± 2.06E+04	3.44E+05 ± 2.53E+05	1.78E+05 ± 9.85E+04	9.63E+04 ± 3.86E+05
	230.586	6.3	3.77E+05 ± 5.07E+04	4.65E+05 ± 1.32E+05	5.41E+05 ± 8.65E+04	5.19E+05 ± 6.88E+04	3.99E+05 ± 1.73E+05
	230.990	0.5	1.99E+05 ± 3.32E+04	2.78E+05 ± 2.55E+05	8.38E+05 ± 3.47E+05	7.07E+05 ± 8.42E+05	1.05E+06 ± 8.10E+05
	231.084	0.5	4.90E+06 ± 7.04E+05	5.73E+06 ± 1.33E+06	2.99E+06 ± 8.03E+05	4.87E+06 ± 1.12E+06	7.02E+06 ± 3.49E+06
(leiokinine A +H)+	232.133	5.1	2.07E+06 ± 2.53E+05	2.19E+06 ± 2.01E+05	2.21E+06 ± 6.09E+05	2.40E+06 ± 4.69E+05	3.00E+06 ± 2.42E+06
	240.123	0.5	6.15E+06 ± 4.62E+05	5.07E+06 ± 6.85E+05	5.88E+06 ± 6.75E+05	4.66E+06 ± 4.73E+04	5.76E+06 ± 1.88E+06
	241.180	6.7	ND	1.58E+06 ± 5.74E+05	3.42E+05 ± 1.12E+04	1.44E+06 ± 2.27E+05	ND
	242.983	0.5	3.31E+05 ± 3.71E+04	4.42E+05 ± 2.42E+05	1.05E+06 ± 3.27E+05	3.98E+05 ± 1.52E+05	1.04E+06 ± 8.40E+05
	244.052	5	5.15E+05 ± 7.57E+04	6.36E+05 ± 1.24E+05	7.63E+04 ± 9.01E+02	6.91E+05 ± 2.21E+05	8.45E+05 ± 6.40E+05
(columbianetin/ marmesin/ nodakenetin +H)+	247.096	6.4	ND	1.23E+06 ± 3.60E+05	1.35E+05 ± 1.03E+05	6.30E+05 ± 1.30E+05	ND
	255.098	0.4	1.03E+06 ± 2.08E+05	5.33E+05 ± 2.76E+05	6.46E+05 ± 2.40E+05	6.63E+05 ± 3.86E+05	4.95E+05 ± 2.88E+05
	256.191	3.2	3.41E+06 ± 7.04E+05	3.28E+06 ± 1.24E+06	5.08E+06 ± 9.48E+04	4.29E+06 ± 1.04E+06	3.65E+06 ± 1.79E+06
	256.300	9.1	9.90E+05 ± 3.81E+04	9.76E+05 ± 8.15E+03	1.53E+06 ± 9.49E+04	1.87E+06 ± 1.82E+05	1.52E+06 ± 1.25E+06
	260.222	1.1	1.07E+07 ± 1.34E+06	1.28E+07 ± 6.43E+06	1.63E+07 ± 3.27E+06	1.23E+07 ± 5.26E+06	1.73E+07 ± 8.76E+06
	260.259	6.2	9.49E+05 ± 1.91E+05	9.08E+05 ± 1.57E+05	1.22E+06 ± 1.04E+05	9.46E+05 ± 3.08E+05	1.39E+06 ± 8.83E+06
	263.565	4.2	1.35E+05 ± 2.79E+04	1.73E+05 ± 5.60E+04	1.96E+05 ± 6.26E+04	1.72E+05 ± 1.52E+04	2.00E+05 ± 6.60E+05
	272.222	4.1	4.94E+06 ± 1.53E+05	7.73E+06 ± 3.99E+06	8.09E+06 ± 4.12E+06	9.23E+06 ± 6.32E+06	8.39E+06 ± 4.56E+06
	274.201	1	5.73E+05 ± 1.43E+05	7.15E+05 ± 6.38E+05	1.09E+06 ± 3.80E+05	6.29E+05 ± 5.00E+05	1.08E+06 ± 4.08E+06
	274.238	4	2.13E+06 ± 1.48E+05	2.60E+06 ± 1.48E+06	3.22E+06 ± 9.31E+05	2.84E+06 ± 1.85E+06	3.55E+06 ± 1.38E+06
(cyanidin 3-O-(6"-	279.048	0.3	1.23E+05	1.72E+05	1.64E+05	1.75E+05	1.72E+05

malonyl-glucoside) +Na)$^+$			± 1.72E+04	± 2.84E+04	± 5.96E+04	± 2.59E+04	± 1.87E+06
	284.331	9.8	1.05E+06 ± 5.72E+04	1.24E+06 ± 1.52E+05	7.13E+07 ± 2.72E+07	1.16E+08 ± 4.32E+07	6.76E+07 ± 3.71E+07
(artemisin/farinosin/dihydrogriesenin/ grosshemin/ helenalin/ mexicanin/ iparthenin/ stramonin/ microhelenin A +Na)$^+$	285.112	0.4	8.50E+04 ± 1.18E+04	2.42E+05 ± 5.28E+04	1.81E+05 ± 1.15E+05	2.89E+05 ± 1.74E+05	1.70E+05 ± 3.71E+07
	285.335	9.8	1.96E+05 ± 1.50E+04	2.39E+05 ± 3.45E+04	1.47E+07 ± 5.57E+06	2.40E+07 ± 8.88E+06	1.40E+07 ± 7.59E+06
(buchananine +H)$^+$	286.092	1.6	2.38E+06 ± 6.04E+05	3.11E+06 ± 2.70E+06	5.56E+06 ± 1.08E+06	1.84E+06 ± 1.14E+06	3.32E+06 ± 5.89E+06
(buchananine +H)$^+$	286.092	2.4	1.22E+06 ± 3.40E+05	1.58E+06 ± 1.37E+06	2.71E+06 ± 5.08E+05	8.98E+05 ± 6.31E+05	1.57E+06 ± 1.06E+06
	286.238	5	2.41E+06 ± 2.08E+05	2.78E+06 ± 1.16E+06	3.48E+06 ± 1.01E+06	2.99E+06 ± 1.48E+06	3.81E+06 ± 1.27E+06
	287.980	0.5	1.77E+05 ± 1.67E+04	2.24E+05 ± 1.23E+05	6.65E+05 ± 1.83E+05	4.92E+05 ± 4.77E+05	6.19E+05 ± 1.80E+06
	288.253	5	5.81E+06 ± 3.39E+05	7.17E+06 ± 3.42E+06	9.02E+06 ± 2.97E+06	7.11E+06 ± 4.01E+06	9.08E+06 ± 4.65E+06
	289.993	0.3	2.73E+05 ± 7.29E+04	5.76E+05 ± 1.00E+05	4.28E+05 ± 5.38E+04	5.91E+05 ± 1.45E+05	3.80E+05 ± 4.77E+06
	293.055	0.5	2.02E+05 ± 1.14E+04	2.13E+05 ± 4.25E+04	1.01E+05 ± 2.50E+04	1.71E+05 ± 3.68E+04	2.49E+05 ± 1.24E+05
	294.183	4.3	2.33E+05 ± 3.01E+04	3.05E+05 ± 1.66E+05	4.58E+05 ± 2.08E+05	2.88E+05 ± 1.60E+05	3.38E+05 ± 6.67E+04
	295.086	0.5	1.53E+05 ± 2.61E+04	1.46E+05 ± 2.40E+04	7.36E+04 ± 1.38E+04	1.25E+05 ± 2.89E+04	1.64E+05 ± 9.72E+04
(buchananine +Na)$^+$	308.074	1.6	6.79E+05 ± 2.03E+05	8.73E+05 ± 7.78E+05	1.56E+06 ± 2.96E+05	4.67E+05 ± 2.75E+05	9.18E+05 ± 4.29E+05
(buchananine +Na)$^+$	308.074	2.4	2.62E+05 ± 7.98E+04	3.34E+05 ± 2.71E+05	5.69E+05 ± 6.09E+04	1.82E+05 ± 1.17E+05	3.46E+05 ± 3.38E+05
(dihydrocapsaicin +H)$^+$	308.222	5.3	2.99E+05 ± 2.86E+04	3.26E+05 ± 1.68E+05	5.70E+05 ± 2.29E+05	4.04E+05 ± 3.45E+05	5.71E+05 ± 1.43E+05
	312.129	0.35	1.74E+06 ± 6.29E+04	2.47E+06 ± 4.00E+05	1.91E+06 ± 4.65E+05	2.48E+06 ± 7.48E+05	2.06E+06 ± 8.25E+05
	312.363	10.6	2.36E+07 ± 1.52E+05	2.36E+07 ± 8.81E+04	2.03E+07 ± 9.63E+06	8.78E+06 ± 6.92E+05	1.48E+07 ± 9.62E+06
	327.101	0.4	5.01E+05 ± 7.13E+04	6.76E+05 ± 1.90E+05	6.75E+04 ± 1.86E+04	6.29E+05 ± 2.69E+05	6.58E+05 ± 1.02E+07
(3',5'-cyclic dGMP/2',3'-cyclic AMP/3',5'-cyclic AMP +H)$^+$	330.059	0.5	3.06E+06 ± 4.83E+05	2.98E+06 ± 7.18E+05	3.58E+06 ± 7.30E+05	2.74E+06 ± 4.28E+05	4.59E+06 ± 2.16E+06
	331.111	0.3	1.03E+06 ± 1.09E+05	1.25E+06 ± 1.70E+05	3.63E+06 ± 5.12E+06	8.56E+05 ± 1.06E+05	7.53E+05 ± 2.11E+06
	332.331	9.8	7.67E+06 ± 6.67E+05	5.63E+06 ± 1.67E+05	6.97E+06 ± 1.88E+06	2.60E+07 ± 3.47E+07	5.99E+06 ± 2.87E+06
	337.105	9.8	1.47E+06 ± 2.51E+04	1.54E+06 ± 1.11E+05	1.31E+06 ± 1.30E+05	1.07E+06 ± 5.03E+04	1.42E+06 ± 2.50E+06
(portulacaxanthin I +Na)$^+$	347.085	0.3	1.99E+06 ± 9.17E+04	2.71E+06 ± 3.58E+05	2.11E+06 ± 3.62E+05	2.36E+06 ± 6.74E+04	2.38E+06 ± 5.63E+05
(5'-adenosine monophosphate/2'-deoxyguanosine 5'-monophosphate +H)$^+$	348.070	0.6	9.18E+05 ± 1.26E+05	6.73E+05 ± 2.11E+05	6.43E+05 ± 1.62E+05	5.62E+05 ± 3.17E+04	5.26E+05 ± 1.04E+06

	355.175	6.3	1.00E+03 ± 0.00E+00	8.53E+05 ± 8.20E+05	5.60E+04 ± 4.31E+04	1.59E+05 ± 1.71E+04	1.33E+03 ± 3.16E+05
	357.075	4.7	3.27E+05 ± 1.98E+04	3.75E+05 ± 2.89E+04	1.88E+05 ± 1.46E+04	4.04E+05 ± 5.70E+04	3.41E+05 ± 1.89E+05
	357.075	5.1	5.59E+05 ± 1.04E+04	6.36E+05 ± 7.65E+03	3.46E+05 ± 3.38E+04	7.12E+05 ± 1.73E+05	7.12E+05 ± 2.06E+05
(miraxanthin-I +H)$^+$	359.091	4.7	1.82E+06 ± 1.35E+05	2.11E+06 ± 2.74E+05	1.03E+06 ± 8.30E+04	2.28E+06 ± 2.44E+05	2.05E+06 ± 7.45E+05
	364.176	2.6	7.64E+05 ± 2.53E+05	1.04E+06 ± 3.38E+05	1.03E+06 ± 5.31E+05	8.32E+05 ± 1.18E+05	1.63E+06 ± 3.27E+05
	366.133	0.6	5.78E+05 ± 9.82E+04	8.10E+05 ± 1.28E+05	6.42E+05 ± 7.21E+04	6.09E+05 ± 5.15E+04	8.61E+05 ± 4.67E+05
	380.149	1.2	8.96E+04 ± 1.62E+04	9.36E+04 ± 1.89E+04	1.23E+06 ± 3.98E+05	2.03E+06 ± 8.47E+05	2.09E+06 ± 6.81E+05
(chaparrin +H)$^+$	381.191	6.3	4.06E+06 ± 4.17E+05	5.38E+06 ± 1.14E+06	6.32E+06 ± 7.85E+05	5.00E+06 ± 8.42E+05	4.27E+06 ± 1.25E+06
	388.248	6.6	2.58E+05 ± 1.01E+05	2.27E+05 ± 1.18E+05	5.68E+05 ± 1.47E+05	2.82E+05 ± 1.32E+05	8.15E+05 ± 2.00E+06
	390.212	6.4	ND	3.13E+06 ± 5.35E+05	3.06E+05 ± 2.17E+04	1.75E+06 ± 4.97E+05	ND
	394.059	5.5	2.48E+06 ± 2.36E+05	3.08E+06 ± 4.57E+05	6.93E+05 ± 1.88E+05	3.24E+06 ± 4.33E+05	3.99E+06 ± 2.21E+06
	394.059	6.2	9.16E+05 ± 7.41E+04	1.06E+06 ± 2.86E+05	2.18E+05 ± 6.88E+04	1.14E+06 ± 2.41E+05	1.59E+06 ± 1.37E+06
	395.168	6.4	ND	7.97E+06 ± 1.43E+06	6.53E+05 ± 2.48E+04	4.69E+06 ± 1.26E+06	ND
	396.075	5.5	1.01E+07 ± 1.10E+06	1.24E+07 ± 1.76E+06	2.68E+06 ± 7.28E+05	1.32E+07 ± 1.73E+06	1.62E+07 ± 8.94E+06
(echimidine/ heliosupine +H)$^+$	398.217	6.8	2.28E+05 ± 3.63E+04	5.05E+05 ± 7.87E+04	1.12E+06 ± 7.65E+05	4.86E+05 ± 2.24E+05	2.36E+05 ± 8.82E+06
	401.259	4.6	3.09E+05 ± 1.61E+05	5.57E+05 ± 3.58E+05	1.28E+06 ± 9.35E+05	6.64E+05 ± 5.06E+04	1.44E+06 ± 6.89E+05
	402.249	4.1	9.13E+05 ± 7.60E+04	1.38E+06 ± 1.17E+06	2.33E+06 ± 1.10E+06	1.43E+06 ± 1.20E+06	1.73E+06 ± 2.76E+05
(chaparrin +Na)$^+$	403.173	6.8	3.62E+05 ± 1.06E+04	7.29E+05 ± 8.21E+04	1.60E+06 ± 9.93E+05	7.36E+05 ± 3.46E+05	3.76E+05 ± 7.57E+05
(lobinaline N-oxide +H)$^+$	403.274	2.6	3.77E+05 ± 1.63E+05	6.98E+05 ± 3.23E+05	1.15E+06 ± 7.00E+05	6.61E+05 ± 1.19E+05	8.79E+05 ± 4.83E+05
(lobinaline N-oxide +H)$^+$	403.274	4.3	2.02E+05 ± 4.85E+04	2.94E+05 ± 1.11E+05	6.07E+05 ± 4.24E+05	3.19E+05 ± 2.06E+04	4.44E+05 ± 4.61E+05
	406.280	4.6	7.82E+05 ± 9.68E+04	1.16E+06 ± 9.59E+05	1.69E+06 ± 4.93E+05	9.87E+05 ± 5.73E+05	1.52E+06 ± 6.12E+05
	407.116	0.5	5.70E+06 ± 1.94E+06	9.35E+06 ± 6.24E+06	9.37E+06 ± 4.79E+06	5.97E+06 ± 3.88E+06	1.06E+07 ± 5.13E+06
	407.123	0.7	1.72E+06 ± 3.12E+05	2.54E+06 ± 1.82E+06	3.43E+06 ± 1.88E+06	2.33E+06 ± 1.06E+06	2.80E+06 ± 4.45E+06
	408.075	5.9	4.37E+05 ± 1.45E+05	3.78E+05 ± 9.83E+04	7.50E+04 ± 3.28E+04	4.16E+05 ± 1.89E+05	8.07E+05 ± 1.13E+06
	408.075	6.5	5.94E+05 ± 2.05E+05	5.81E+05 ± 2.83E+05	1.23E+05 ± 6.14E+04	5.86E+05 ± 3.33E+05	1.18E+06 ± 3.97E+05
	410.090	5.9	1.67E+06 ± 5.46E+05	1.51E+06 ± 4.25E+05	2.99E+05 ± 1.38E+05	1.60E+06 ± 6.89E+05	3.04E+06 ± 1.22E+06
	412.193	0.5	2.66E+05 ± 2.48E+04	3.80E+05 ± 2.15E+05	5.81E+05 ± 4.62E+05	5.78E+05 ± 6.09E+05	6.71E+05 ± 1.43E+06
	416.228	6.3	4.09E+06 ± 4.94E+05	4.97E+06 ± 6.45E+05	5.75E+06 ± 2.59E+05	4.58E+06 ± 5.70E+05	4.01E+06 ± 1.88E+06
	416.264	4.9	1.98E+05 ± 2.70E+04	2.49E+05 ± 1.73E+05	3.49E+05 ± 8.76E+04	2.81E+05 ± 2.01E+05	3.42E+05 ± 2.05E+06
	418.057	5.5	1.01E+06 ± 1.14E+05	1.25E+06 ± 1.96E+05	2.53E+05 ± 7.90E+04	1.30E+06 ± 1.63E+05	1.55E+06 ± 6.78E+05
	418.280	5.2	1.71E+05	2.90E+05	4.14E+05	4.17E+05	4.52E+05

			± 4.12E+04	± 1.90E+05	± 2.99E+05	± 3.59E+05	± 6.25E+05
	420.295	5.2	1.63E+06 ± 4.41E+05	1.84E+06 ± 1.15E+06	3.28E+06 ± 3.45E+05	1.83E+06 ± 9.14E+05	2.63E+06 ± 1.23E+06
	421.084	1.7	4.39E+05 ± 5.34E+04	3.32E+05 ± 4.62E+04	8.06E+05 ± 3.67E+05	2.78E+05 ± 9.40E+04	2.56E+05 ± 1.33E+06
(braxin C +Na)$^{+}$	421.183	6.3	6.99E+06 ± 5.87E+05	8.36E+06 ± 1.37E+06	9.83E+06 ± 1.38E+05	7.85E+06 ± 1.03E+06	6.73E+06 ± 3.60E+06
	422.177	0.4	2.08E+06 ± 3.02E+05	3.23E+06 ± 5.57E+05	2.43E+06 ± 8.37E+05	3.29E+06 ± 1.22E+06	2.58E+06 ± 2.40E+06
	423.090	0.5	3.99E+06 ± 1.03E+06	6.55E+06 ± 4.37E+06	6.55E+06 ± 2.72E+06	5.00E+06 ± 2.22E+06	7.90E+06 ± 3.16E+06
	424.069	6.3	8.90E+05 ± 5.39E+04	8.45E+05 ± 4.40E+05	1.34E+05 ± 3.74E+04	4.51E+05 ± 1.91E+05	6.34E+05 ± 4.15E+06
	425.092	6.1	2.27E+06 ± 1.65E+05	2.18E+06 ± 6.55E+04	2.41E+06 ± 3.67E+05	1.72E+06 ± 1.20E+05	1.82E+06 ± 7.21E+05
	428.212	0.6	2.22E+05 ± 4.70E+04	2.50E+05 ± 1.22E+05	4.69E+05 ± 4.45E+05	3.47E+05 ± 2.46E+05	4.51E+05 ± 7.54E+05
	429.105	0.7	3.06E+05 ± 6.69E+04	4.38E+05 ± 2.65E+05	4.87E+05 ± 2.93E+05	3.84E+05 ± 1.36E+05	4.32E+05 ± 3.71E+04
	429.105	1.4	4.56E+05 ± 1.18E+05	7.14E+05 ± 5.14E+05	8.23E+05 ± 3.17E+05	5.04E+05 ± 1.87E+05	6.91E+05 ± 1.54E+05
	430.192	0.5	1.86E+05 ± 5.72E+04	3.57E+05 ± 2.99E+05	3.63E+05 ± 3.06E+05	2.23E+05 ± 1.58E+05	3.37E+05 ± 2.05E+05
	430.192	2.1	1.94E+06 ± 2.56E+05	2.53E+06 ± 9.17E+05	3.63E+06 ± 1.90E+06	2.08E+06 ± 4.28E+05	2.55E+06 ± 1.22E+06
	432.171	0.4	5.44E+06 ± 1.76E+06	1.37E+07 ± 1.23E+06	8.84E+06 ± 3.81E+06	1.52E+07 ± 6.97E+06	8.25E+06 ± 4.08E+06
	442.091	4.8	5.30E+05 ± 1.04E+05	4.90E+05 ± 2.46E+05	4.94E+05 ± 2.27E+04	2.95E+05 ± 8.62E+04	2.93E+05 ± 5.09E+06
	446.187	0.4	6.03E+05 ± 1.42E+05	8.99E+05 ± 1.18E+04	7.04E+05 ± 2.71E+05	9.96E+05 ± 2.80E+05	7.73E+05 ± 3.24E+05
	448.166	0.4	4.29E+06 ± 1.20E+06	6.52E+06 ± 2.85E+05	5.03E+06 ± 1.88E+06	6.57E+06 ± 1.98E+06	4.18E+06 ± 1.93E+06
	451.174	0.4	5.00E+06 ± 3.43E+05	3.82E+06 ± 6.47E+05	4.77E+06 ± 1.72E+06	3.84E+06 ± 2.30E+05	4.16E+06 ± 7.57E+05
	461.163	5.1	2.77E+06 ± 3.12E+05	2.48E+06 ± 5.83E+05	4.06E+06 ± 1.85E+06	3.25E+06 ± 8.86E+05	4.28E+06 ± 7.84E+05
	469.134	6	3.25E+06 ± 3.65E+05	3.67E+06 ± 1.31E+06	4.67E+06 ± 5.26E+05	3.11E+06 ± 3.84E+05	3.66E+06 ± 6.87E+05
	469.134	4.2	2.04E+06 ± 4.48E+05	2.42E+06 ± 7.96E+05	2.74E+06 ± 9.03E+05	2.42E+06 ± 2.17E+05	3.06E+06 ± 5.33E+05
	471.222	7	ND	6.58E+05 ± 3.90E+05	1.11E+05 ± 9.80E+04	5.67E+05 ± 4.27E+04	ND
	471.222	7.7	ND	4.51E+05 ± 1.83E+05	7.90E+04 ± 1.08E+04	4.27E+05 ± 8.41E+04	ND
(isorhamnetin 3-O-glucoside/ isorhamnetin 7-O-rhamnoside +H)$^{+}$	479.118	6.3	3.88E+05 ± 5.72E+04	6.14E+05 ± 8.40E+04	7.54E+05 ± 4.46E+05	8.76E+05 ± 3.21E+05	7.15E+05 ± 4.38E+05
(isorhamnetin 3-O-glucoside/ isorhamnetin 7-O-rhamnoside +H)$^{+}$	479.118	6	6.05E+05 ± 4.02E+05	1.29E+06 ± 1.18E+06	1.67E+06 ± 1.55E+06	2.28E+06 ± 7.23E+05	1.76E+06 ± 1.02E+06
	483.111	0.5	3.27E+05 ± 1.09E+05	6.22E+05 ± 4.22E+05	5.12E+05 ± 2.68E+05	4.31E+05 ± 3.12E+05	6.87E+05 ± 1.01E+06
	484.218	7.1	9.84E+05 ± 7.78E+04	1.88E+06 ± 3.70E+05	2.10E+06 ± 7.21E+05	1.61E+06 ± 1.68E+05	9.29E+05 ± 1.73E+05
	489.173	7.1	1.04E+06 ± 9.07E+04	1.82E+06 ± 3.66E+05	2.07E+06 ± 7.16E+05	1.59E+06 ± 1.75E+05	9.58E+05 ± 1.15E+05
	522.203	0.4	2.26E+07 ± 9.38E+05	1.47E+07 ± 3.41E+06	1.87E+07 ± 4.82E+06	1.86E+07 ± 9.58E+06	2.07E+07 ± 1.09E+07

	525.117	4.6	2.32E+06 ± 1.46E+05	2.24E+06 ± 9.47E+05	4.13E+05 ± 1.13E+05	1.43E+06 ± 3.15E+05	1.87E+06 ± 1.04E+07
	526.221	6.3	4.83E+04 ± 5.58E+04	3.93E+05 ± 1.35E+05	3.46E+05 ± 2.19E+05	2.77E+05 ± 7.53E+04	1.08E+05 ± 1.20E+06
	526.221	6.5	1.14E+05 ± 9.96E+04	1.51E+06 ± 5.07E+05	8.24E+05 ± 6.90E+05	7.29E+05 ± 1.43E+05	2.69E+05 ± 1.52E+05
	540.201	5.5	5.05E+05 ± 1.07E+05	9.12E+05 ± 2.59E+05	1.15E+06 ± 1.92E+05	1.38E+06 ± 1.10E+05	7.48E+05 ± 3.03E+05
	557.221	6	ND	2.60E+06 ± 1.00E+06	2.92E+05 ± 4.72E+04	1.61E+06 ± 1.24E+05	ND
	560.270	6.3	1.53E+05 ± 3.55E+04	3.40E+05 ± 9.23E+04	7.61E+05 ± 8.78E+05	3.44E+05 ± 1.27E+05	1.88E+05 ± 1.05E+05
	589.069	0.5	1.22E+05 ± 2.82E+04	1.44E+05 ± 7.05E+04	3.81E+05 ± 1.09E+05	2.78E+05 ± 2.53E+05	4.39E+05 ± 3.37E+05
	594.224	0.4	1.57E+06 ± 2.15E+05	3.22E+06 ± 8.46E+05	2.02E+06 ± 9.04E+05	3.72E+06 ± 5.94E+05	2.41E+06 ± 1.40E+06
	628.110	0.3	1.13E+06 ± 1.02E+05	6.01E+05 ± 2.93E+05	7.56E+05 ± 2.84E+05	6.65E+05 ± 5.57E+05	8.89E+05 ± 1.20E+06
	638.214	0.4	1.36E+06 ± 1.41E+05	9.00E+05 ± 1.02E+05	1.23E+06 ± 2.07E+05	1.07E+06 ± 4.26E+05	1.34E+06 ± 3.31E+05
	644.084	0.3	1.01E+06 ± 4.69E+04	5.79E+05 ± 2.22E+05	8.84E+05 ± 2.83E+05	7.79E+05 ± 6.33E+05	1.15E+06 ± 2.26E+05
	645.144	6.5	7.47E+05 ± 9.87E+04	7.68E+05 ± 8.64E+05	8.00E+05 ± 1.82E+05	2.19E+05 ± 1.95E+05	4.72E+05 ± 5.65E+05
	651.245	0.3	3.40E+07 ± 9.39E+05	2.98E+07 ± 1.08E+07	2.10E+07 ± 6.19E+06	3.54E+07 ± 9.87E+06	3.13E+07 ± 1.70E+07
	664.460	4.7	3.70E+05 ± 4.46E+04	4.04E+05 ± 1.93E+05	6.33E+05 ± 2.38E+05	4.72E+05 ± 1.28E+05	6.04E+05 ± 1.69E+07
	701.143	4.6	4.39E+05 ± 2.22E+04	5.62E+05 ± 1.68E+05	4.06E+04 ± 6.74E+03	7.50E+05 ± 1.80E+05	8.28E+05 ± 2.62E+05
	703.280	6.9	3.14E+04 ± 1.33E+04	8.04E+04 ± 6.51E+04	2.43E+05 ± 3.37E+05	5.20E+04 ± 3.65E+04	5.76E+04 ± 4.76E+05
	711.183	0.3	7.02E+05 ± 9.00E+04	4.56E+05 ± 1.28E+05	4.31E+05 ± 1.23E+05	4.46E+05 ± 1.78E+05	5.70E+05 ± 3.08E+05
	756.277	0.4	5.83E+05 ± 8.93E+04	1.53E+06 ± 2.80E+05	9.01E+05 ± 4.10E+05	1.88E+06 ± 7.88E+05	9.55E+05 ± 3.93E+05
	773.155	6.3	4.11E+06 ± 2.06E+05	4.58E+06 ± 9.52E+05	5.83E+06 ± 7.38E+05	3.69E+06 ± 1.05E+06	4.94E+06 ± 2.27E+06
	780.320	5.8	3.52E+05 ± 6.99E+04	5.64E+05 ± 2.39E+05	5.92E+05 ± 1.72E+05	6.23E+05 ± 1.75E+05	5.23E+05 ± 2.48E+06
	780.570	5.8	3.97E+05 ± 5.68E+04	5.74E+05 ± 2.41E+05	6.06E+05 ± 1.84E+05	6.88E+05 ± 1.80E+05	5.66E+05 ± 6.98E+04
	809.529	4.6	2.25E+06 ± 4.02E+05	2.78E+06 ± 1.46E+06	5.28E+06 ± 3.15E+06	3.62E+06 ± 4.90E+05	4.26E+06 ± 2.37E+06
	814.282	0.4	1.33E+06 ± 5.12E+04	1.82E+06 ± 1.77E+05	1.69E+06 ± 5.04E+05	2.25E+06 ± 5.91E+05	1.78E+06 ± 1.85E+06
	833.140	6.1	8.23E+05 ± 1.10E+05	7.68E+05 ± 9.64E+05	9.42E+05 ± 5.09E+05	1.42E+05 ± 1.49E+05	5.97E+05 ± 8.91E+05
	835.156	6.1	2.01E+07 ± 2.52E+06	1.81E+07 ± 2.23E+07	2.14E+07 ± 1.25E+07	3.62E+06 ± 4.07E+06	1.38E+07 ± 1.48E+07
	853.478	4.6	7.47E+05 ± 1.45E+05	8.84E+05 ± 4.60E+05	1.39E+06 ± 7.57E+05	1.09E+06 ± 1.06E+05	1.28E+06 ± 1.46E+07
	857.138	6.1	1.51E+06 ± 1.31E+05	1.20E+06 ± 1.34E+06	1.53E+06 ± 8.88E+05	2.94E+05 ± 3.21E+05	9.54E+05 ± 9.30E+05
	885.356	0.4	1.29E+06 ± 1.32E+05	1.64E+06 ± 4.03E+05	1.14E+06 ± 2.30E+05	1.85E+06 ± 2.73E+05	1.40E+06 ± 8.95E+05
	918.330	0.4	3.68E+05 ± 4.06E+04	8.32E+05 ± 1.93E+04	5.82E+05 ± 2.04E+05	9.89E+05 ± 4.14E+05	5.81E+05 ± 4.94E+05
	961.223	5.6	4.60E+05 ± 1.95E+05	5.94E+05 ± 2.98E+05	7.29E+05 ± 2.60E+05	5.78E+05 ± 2.34E+05	8.38E+05 ± 2.18E+05
	976.332	0.4	4.28E+05	6.20E+05	5.74E+05	7.22E+05	6.19E+05

			± 3.81E+04	± 1.88E+04	± 1.37E+05	± 2.57E+05	± 1.77E+05
	1031.326	0.4	2.57E+06 ± 4.32E+05	1.57E+06 ± 6.85E+05	1.61E+06 ± 1.02E+06	1.87E+06 ± 1.18E+06	1.66E+06 ± 6.05E+05
	1099.320	0.4	1.16E+06 ± 8.89E+04	1.40E+06 ± 5.22E+05	1.26E+06 ± 4.18E+05	1.51E+06 ± 1.91E+05	9.91E+05 ± 4.41E+05

Table S2. Putative metabolites identified across 15/15 samples in elicitation experiments. Metabolite categories included are nitrogen-containing secondary metabolites, phenolics, and amino acids.

Putative metabolite	m/z	Chemical formula	KEGG ID	Metabolite class and/or subclass
(-)-epigallocatechin	306.0740	$C_{15}H_{14}O_7$	C12136	flavanol
(+)-galbacin	340.1311	$C_{20}H_{20}O_5$	C10616	phenylpropanoid-lignan
(+)-gallocatechin	306.0740	$C_{15}H_{14}O_7$	C12127	flavanol
13-hydroxylupanine	264.1838	$C_{15}H_{24}N_2O_2$	C02621	alkaloid-quinolizidine
3-hydroxystachydrine	159.0895	$C_7H_{13}NO_3$	C10151	alkaloid-pyrrolidine
3-O-methylbatatasin II	258.1256	$C_{16}H_{18}O_3$	C10271	bibenzyl
3,4-DHPEA-EDA	320.1260	$C_{17}H_{20}O_6$	n/a	polyphenol
5-hydroxyconiferyl alcohol	196.0736	$C_{10}H_{12}O_4$	C12205	phenylpropanoid-monolignol
5-hydroxyferulic acid	210.0528	$C_{10}H_{10}O_5$	C05619	phenylpropanoid-monolignol
7-hydroxysecoisolariciresinol	378.1679	$C_{20}H_{26}O_7$	-	phenylpropanoid-lignan
8-prenylnaringenin	340.1311	$C_{20}H_{20}O_5$	C18023	flavonoid
alexine	189.1001	$C_8H_{15}NO_4$	C10124	alkaloid-pyrrolizidine
ammothamnine	264.1838	$C_{15}H_{24}N_2O_2$	C10749	alkaloid-quinolizidine
anaferine	224.1889	$C_{13}H_{24}N_2O$	C06183	alkaloid-piperidine
anatoxin A	165.1154	$C_{10}H_{15}NO$	C10841	alkaloid-tropane
apigenin 6,8-di-C-glucoside	594.1585	$C_{27}H_{30}O_{15}$	C10195	flavone
apigenin 7-O-diglucuronide	622.1170	$C_{27}H_{26}O_{17}$	n/a	flavone
arecoline	155.0950	$C_8H_{13}NO_2$	C10129	alkaloid-pyridine
arginine	174.1117	$C_4H_{14}N_4O_2$	C00062	amino acid
asparagine	132.0530	$C_4H_8N_2O_3$	C00152	amino acid
australine	189.1001	$C_8H_{15}NO_4$	C10132	alkaloid-pyrrolizidine
buchananine	285.0849	$C_{12}H_{15}NO_7$	C10134	alkaloid-piperidine
caffeic aldehyde	164.0473	$C_9H_8O_3$	C10945	phenylpropanoid-monolignol
calystegin A3	159.0895	$C_7H_{13}NO_3$	C10850	alkaloid-tropane
capsaicin	305.1991	$C_{18}H_{27}NO_3$	C06866	amide
carnegine	221.1416	$C_{13}H_{19}NO_2$	C09375	alkaloid-isoquinoline
cassythine	342.1340	$C_{19}H_{19}NO_5$	C09380	alkaloid-isoquinoline
castanospermine	189.1001	$C_8H_{15}NO_4$	C02256	alkaloid-Indolizidine
chlorogenic acid	354.0951	$C_{16}H_{18}O_9$	C00852	phenylpropanoid-monolignol
cichoriin	340.0794	$C_{15}H_{16}O_9$	C09206	phenylpropanoid-coumarin
cochlearine	261.1365	$C_{15}H_{19}NO_3$	C10853	alkaloid-tropane
columbianetin	246.0892	$C_{14}H_{14}O_4$	C09210	phenylpropanoid-coumarin
coniferin	342.1315	$C_{16}H22O_8$	C00761	phenylpropanoid-monolignol
coumaric acid	164.0473	$C_9H_8O_3$	C00811	phenylpropanoid-monolignol
cuscohygrine	224.1889	$C_{13}H_{24}N_2O$	C06521	alkaloid-pyrrolidine
cyanidin 3-O-(6''-caffeoyl-glucoside)	611.1401	$C_{30}H_{27}O_{14}$	C16369	flavonoid-anthocyanidin
cyanidin 3-O-(6''-malonyl-glucoside)	535.1088	$C_{24}H_{23}O_{14}$	C12643	flavonoid-anthocyanidin
cyclolariciresinol	360.1573	$C_{20}H_{24}O_6$	n/a	phenylpropanoid-lignan
darlingine	219.1259	$C_{13}H_{17}NO_2$	C10857	alkaloid-tropane
decuroside III	570.1749	$C_{26}H_{34}O_{14}$	C09257	phenylpropanoid-coumarin
deidaclin	271.1056	$C_{12}H_{17}NO_6$	C08329	cyanogenic glucoside
delphinidin 3-O-(6''-p-coumaroyl-glucoside	611.1401	$C_{30}H_{27}O_{14}$	C16370	flavonoid-anthocyanidin

Putative metabolite	m/z	Chemical formula	KEGG ID	Metabolite class and/or subclass
deoxypodophyllotoxin	398.1366	$C_{22}H_{22}O_7$	C10556	phenylpropanoid-lignan
dihydrocapsaicin	307.2147	$C_{18}H_{29}NO_3$	C16952	amide
dioscorine	221.1416	$C_{13}H_{19}NO_2$	C10142	alkaloid-piperidine
diosmin	608.1741	$C_{28}H_{32}O_{15}$	C10039	flavone
echimidine	397.2101	$C_{20}H_{31}NO_7$	C10299	alkaloid-pyrrolizidine
elaeocarpine	257.1416	$C_{16}H_{19}NO_2$	C10591	alkaloid-Indolizidine
elaeokanine C	211.1572	$C_{12}H_{21}NO_2$	C10592	alkaloid-Indolizidine
elemicin	208.1099	$C_{12}H_{16}O_3$	C10451	phenylpropanoid-monolignol
entadamide A	161.0510	$C_{16}H_{11}NO_2S$	C20204	amide
ephedrine	165.1154	$C_{10}H_{15}NO$	C01575	alkaloid
erythroidine	273.1365	$C_{16}H_{19}NO_3$	C06531	alkaloid-isoquinoline
esculin	340.0794	$C_{15}H_{16}O_9$	C09264	phenylpropanoid-coumarin
ferulaldehyde	178.0630	$C_{10}H_{10}O_3$	C02666	phenylpropanoid-monolignol
ferulic acid	194.0579	$C_{10}H_{10}O_4$	C01494	phenylpropanoid-monolignol
fulvine	309.1576	$C_{16}H_{23}NO_5$	C10304	alkaloid-pyrrolizidine
glutamine	146.0691	$C_5H_{10}N_2O_3$	C00064	amino acid
gynocardin	303.0954	$C_{12}H_{17}NO_8$	C08331	cyanogenic glucoside
harzianopyridone	281.1263	$C_{14}H_{19}NO_5$	C10150	alkaloid-piperidine
heliosupine	397.2101	$C_{20}H_{31}NO_7$	C10319	alkaloid-pyrrolizidine
heliotridine	155.0950	$C_8H_{13}NO_2$	C10324	alkaloid-pyrrolizide
herniarin	177.0550	$C_{10}H_8O_3$	C09268	phenylpropanoid-coumarin
hispidulin	300.0634	$C_{16}H_{12}O_6$	C10058	flavone
histidine	155.0690	$C_6H_9N_3O_2$	C00135	amino acid
histidine	155.0695	$C_6H_9N_3O_2$	C00135	amino acid
homostachydrine	157.1103	$C_8H_{15}NO_2$	C08283	alkaloid-pyrrolidine
hordenine	165.1154	$C_{10}H_{15}NO$	C06199	alkaloid
hydroxy-gamma-sanshool	289.2042	$C_{18}H_{27}NO_2$	C17827	amide
hydroxycaffeic acid	196.0372	$C_9H_8O_5$	n/a	phenylpropanoid-monolignol
icaceine	375.2410	$C_{22}H_{33}NO_4$	C08689	terpene alkaloid
indicaxanthin	308.1001	$C_{14}H_{16}N_2O_6$	C08549	betalains
kaempferide	300.0634	$C_{16}H_{12}O_6$	C05903	flavonol
L-citrulline	175.0957	$C_6H_{13}N_3O_3$	C00327	amino acid
lamprolobine	264.1838	$C_{15}H_{24}N_2O_2$	C10769	alkaloid-quinolizidine
lariciresinol	360.1573	$C_{20}H_{24}O_6$	C10646	phenylpropanoid-lignan
leiokinine A	231.1259	$C_{14}H_{17}NO_2$	C10703	alkaloid-quinoline
lentiginosine	157.1103	$C_8H_{15}NO_2$	C10155	alkaloid-Indolizidine
leptodactylone	222.0528	$C_{11}H_{10}O_5$	C09271	phenylpropanoid-coumarin
lobinaline bi-N-oxide	418.2600	$C_{27}H_{34}N_2O_2$	n/a	alkaloid
lobinaline N-oxide	402.2700	$C_{27}H_{34}N_2O$	n/a	alkaloid
lophophorine	235.1208	$C_{13}H_{17}NO_3$	C09573	alkaloid-isoquinoline
lunacrine	273.1365	$C_{16}H_{19}NO_3$	C10711	alkaloid-quinoline
lunularic acid	258.0892	$C_{15}H_{14}O_4$	C10268	bibenzyl

Putative metabolite	m/z	Chemical formula	KEGG ID	Metabolite class and/or subclass
luteolin 7-O-diglucuronide	462.0798	$C_{21}H_{18}O_{12}$	C03515	flavone
luvangetin	258.0892	$C_{15}H_{14}O_4$	C09273	phenylpropanoid-coumarin
magnoshinin	414.2042	$C_{24}H_{30}O_6$	C10658	phenylpropanoid-lignan
marmesin	246.0892	$C_{14}H_{14}O_4$	C09276	phenylpropanoid-coumarin
mellein	178.0630	$C_{10}H_{10}O_3$	n/a	phenylpropanoid-coumarin
methionine	149.0510	$C_5H_{11}NO_2S$	C00073	amino acid
miraxanthin-I	358.0835	$C_{14}H_{18}N_2O_7S$	C08554	betalains
myristicin	192.0786	$C_{11}H_{12}O_3$	C10480	phenylpropanoid-monolignol
N-methylpelletierine	155.1310	$C_9H_{17}NO$	C06184	alkaloid-piperidine
neochlorogenate	354.0951	$C_{16}H_{18}O_9$	C17147	phenylpropanoid-monolignol
neodiosmin	608.1741	$C_{28}H_{32}O_{15}$	n/a	flavone
neoisostegane	414.1679	$C_{23}H_{26}O_7$	C10707	phenylpropanoid-lignan
nitramine	169.1467	$C_{10}H_{19}NO$	C10163	alkaloid-piperidine
nodakenetin	246.0892	$C_{14}H_{14}O_4$	C09278	phenylpropanoid-coumarin
norajmaline	312.1838	$C_{19}H_{24}N_2O_2$	C11810	alkaloid-indole
nordihydrocapsaicin	293.1991	$C_{17}H_{27}NO_3$	C20216	amide
norgalanthamine	273.1365	$C_{16}H_{19}NO_3$	C12173	alkaloid-isoquinoline
nuttaline	264.1838	$C_{15}H_{24}N_2O_2$	C10776	alkaloid-quinolizidine
p-coumaric acid ethyl ester	192.0786	$C_{11}H_{12}O_3$	n/a	phenylpropanoid-monolignol
p-coumaryl alcohol 4-O-glucoside	312.121	$C_{15}H_{20}O_7$	C05855	phenylpropanoid-monolignol
parsonsine	439.2206	$C_{22}H_{33}NO_8$	C10357	alkaloid-pyrrolizidine
peonidin	301.0710	$C_{16}H_{13}O_6^+$	C08726	flavonoid-anthocyanidin
peonidin 3-O-(6''-acetyl-galactoside)	521.1300	$C_{24}H_{25}O_{13}^+$	n/a	flavonoid-anthocyanidin
peonidin 3-O-(6''-acetyl-glucoside)	505.1349	$C_{24}H_{25}O_{12}^+$	n/a	flavonoid-anthocyanidin
peucedanin	258.0892	$C_{15}H_{14}O_4$	C09283	phenylpropanoid-coumarin
phenylalanine	165.0790	$C_9H_{11}NO_2$	C00079	amino acid
portulacaxanthin I	324.0958	$C_{14}H_{16}N_2O_7$	C08564	betalains
pseudoephedrine	165.1154	$C_{10}H_{15}NO$	C02765	alkaloid
retronecine	155.0950	$C_8H_{13}NO_2$	C06177	alkaloid-pyrrolizide
rutarin	424.1369	$C_{20}H_{24}O_{10}$	C09309	phenylpropanoid-coumarin
scopoline	155.0950	$C_8H_{13}NO_2$	C10866	alkaloid-tropane
secoisolariciresinol	362.1729	$C_{20}H_{26}O_6$	C20456	phenylpropanoid-lignan
sinapic acid	224.0685	$C_{11}H_{12}O_5$	C00482	phenylpropanoid-monolignol
sinapoyl malate	340.0794	$C_{15}H_{16}O_9$	C02887	phenylpropanoid-monolignol
theaflavin 3-O-gallate	716.1377	$C_{36}H_{28}O_{16}$	n/a	flavonoid
tryptophan	204.0899	$C_{11}H_{12}N_2O_2$	C00078	amino acid
tyrosine	181.0739	$C_9H_{11}NO_3$	C00082	amino acid
valeroidine	241.1678	$C_{13}H_{23}NO_3$	C10869	alkaloid-tropane
vasicinol	204.0899	$C_{11}H_{12}N_2O_2$	C10743	alkaloid-quinazoline
volkenin	287.1005	$C_{12}H_{17}NO_7$	C08344	cyanogenic glucoside
vulgaxanthin-I	339.1067	$C_{14}H_{17}N_3O_7$	C08568	betalains
yakuchinone A	312.1725	$C_{20}H_{24}O_3$	C20211	diarylheptanoid

Table S3. Putative chemical formula and mass errors for features upregulated due to jasmonic acid elicitation.

m/z	Possible chemical formulae	Mass error (ppm)
195.138	$C_{12}H_{19}O_2^+$	-0.22
241.180	$C_{14}H_{25}O_3^+$	-0.74
289.993	$C_{10}H_2N_4O_7^+$	-4.14
	$C_{12}H_4NO_8^+$	0.49
390.212	$C_{17}H_{26}N_8O_3^+$	0.61
	$C_{18}H_{32}NO_8^+$	0.62
	$C_{16}H_{30}N_4O_7^+$	-2.82
	$C_{19}H_{28}N_5O_4^+$	4.05
395.168	$C_{16}H_{30}N_6O_6^+$	-1.62
	$C_{18}H_{25}N_3O_7^+$	3.03
	$C_{30}H_{21}N^+$	-2.91
471.222	$C_{22}H_{29}N_7O_5^+$	0.99
	$C_{23}H_{35}O_{10}^+$	1.01
	$C_{21}H_{33}N_3O_9^+$	-1.84
	$C_{20}H_{27}N_{10}O_4^+$	-1.86
557.221	$C_{24}H_{35}N_3O_{12}^+$	0.94
	$C_{22}H_{33}N_6O_{11}^+$	-1.47
	$C_{26}H_{37}O_{13}^+$	3.35

Table S4. Putative chemical formula and mass errors for features upregulated due to nanoparticle elicitation. No putative chemical formula was obtained for m/z 284.331 and m/z 285.335, which are therefore excluded from the table.

m/z	Possible chemical formulae	Mass error (ppm)
200.025	$C_{15}H_4O^+$	3.33
256.191	$C_{14}H_{26}NO_3^+$	-1.09
380.149	$C_{21}H_{16}N_8^+$	0.64
	$C_{22}H_{22}NO_5^+$	0.66
	$C_{20}H_{20}N_4O_4^+$	-2.88
	$C_{23}H_{18}N_5O^+$	4.17
